# RGS7 is recurrently mutated in melanoma and promotes migration and invasion of human cancer cells

**DOI:** 10.1038/s41598-017-18851-4

**Published:** 2018-01-12

**Authors:** Nouar Qutob, Ikuo Masuho, Michal Alon, Rafi Emmanuel, Isadora Cohen, Antonella Di Pizio, Jason Madore, Abdel Elkahloun, Tamar Ziv, Ronen Levy, Jared J. Gartner, Victoria K. Hill, Jimmy C. Lin, Yael Hevroni, Polina Greenberg, Alexandra Brodezki, Steven A. Rosenberg, Mickey Kosloff, Nicholas K. Hayward, Arie Admon, Masha Y. Niv, Richard A. Scolyer, Kirill A. Martemyanov, Yardena Samuels

**Affiliations:** 1 Molecular Cell Biology Department, Weizmann Institute of Science, Rehovot, Israel; 2 Department of Neuroscience, The Scripps Research Institute, FL, 33458 USA; 3 Institute of Biochemistry, Food Science and Nutrition, The Robert H. Smith Faculty of Agriculture, Food, and Environment, The Hebrew University, Rehovot, Israel; 4 Melanoma Institute Australia, University of Sydney, NSW, Australia; 5 Tissue Pathology and Diagnostic Oncology, Royal Prince Alfred Hospital, NSW, Australia; 6 National Human Genome Research Institute, US National Institutes of Health, Bethesda, Maryland USA; 7 Department of Biology, Technion-Israel Institute of Technology, Haifa, Israel; 8 National Cancer Institute, Surgery Branch, US National Institutes of Health, Bethesda, Maryland 20892 USA; 9 Department of Human Biology, Faculty of Natural Sciences, University of Haifa, Haifa, Israel; 10 QIMR Berghofer Medical Research Institute, Brisbane, Queensland Australia; 11 Disciplines of Surgery and Pathology, Sydney Medical School, The University of Sydney, Sydney, NSW, Australia

## Abstract

Analysis of 501 melanoma exomes revealed *RGS7*, which encodes a GTPase-accelerating protein (GAP), to be a tumor-suppressor gene. *RGS7* was mutated in 11% of melanomas and was found to harbor three recurrent mutations (p.R44C, p.E383K and p.R416Q). Structural modeling of the most common recurrent mutation of the three (p.R44C) predicted that it destabilizes the protein due to the loss of an H-bond and salt bridge network between the mutated position and the serine and aspartic acid residues at positions 58 as 61, respectively. We experimentally confirmed this prediction showing that the p.R44C mutant protein is indeed destabilized. We further show RGS7 p.R44C has weaker catalytic activity for its substrate Gα_o_, thus providing a dual mechanism for its loss of function. Both of these effects are expected to contribute to loss of function of RGS7 resulting in increased anchorage-independent growth, migration and invasion of melanoma cells. By mutating position 56 in the R44C mutant from valine to cysteine, thereby enabling the formation of a disulfide bridge between the two mutated positions, we slightly increased the catalytic activity and reinstated protein stability, leading to the rescue of RGS7′s function as a tumor suppressor. Our findings identify RGS7 as a novel melanoma driver and point to the clinical relevance of using strategies to stabilize the protein and, thereby, restore its function.

## Introduction

The incidence of melanoma continues to rise globally, at a rate greater than that of any other cancer^1^. Cancer progression is attributed to the acquisition of somatic alterations and, indeed, targeting such mutations with specifically designed drugs has led to significant clinical responses in metastatic melanoma patients^2,3^. Candidate gene analyses are a proven powerful tool for identifying cancer driver genes, such as mutant *BRAF*, the target of the successful FDA-approved inhibitor vemurafenib^4,5^. However, approximately half of melanoma patients do not harbor the *BRAF* mutation, and some patients with this mutation do not respond to the drug, and of those that do, most suffer a relapse within less than 12 months^6–9^. Advances in high-throughput genomic technologies provide an unprecedented opportunity to systematically interrogate the genomic landscape of melanoma and identify new potential targets for treating the disease.

To identify novel mutations that drive melanoma growth, we systematically analyzed somatic mutation data from whole exome/genome sequences of 501 melanomas and searched for alterations that recur at the same chromosomal position in at least four of the samples, as described previously^10^ (Supplementary Table 1). Predictably, the resulting list featured well-documented melanoma drivers, such as known hotspot mutations in *BRAF*, *NRAS*, *RAC1* and *IDH1*^4,11,12^, confirming the validity of our strategy (Supplementary Table 2).

**Figure 1:**
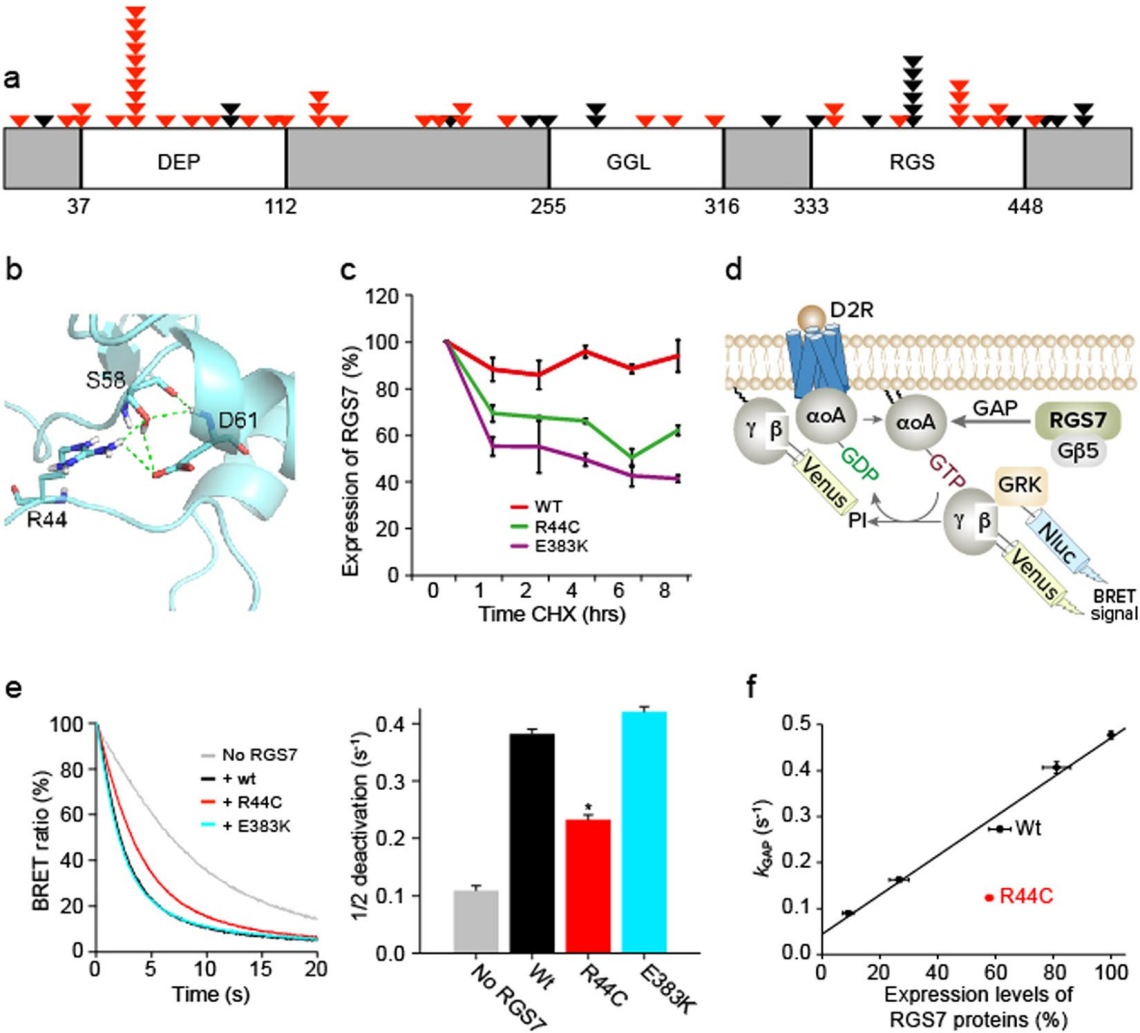
Effects of RGS7 mutation on RGS7 stability and activity. (**a**) The Human RGS7 protein, with conserved domains indicated as blocks, including the Dishevelled domain (DEP); G Protein Gamma-like domain (GGL); RGS domain (RGS). Somatic mutations indicated with arrows. Red triangles indicate deleterious mutations. (**b**) Three dimensional structure of RGS7 N-Terminus, predicting that R44 is involved in an H-bond network with S58 and D61. (**c**) Cells expressing wild-type or mutant RGS7 were treated with cycloheximide (CHX), collected at different time points and then immunoblotted with anti-FLAG antibody. Anti-Cyclin D1 was used as a control and anti-GAPDH was used for normalization. (**d**) A Schematic representation of the BRET-based assay to monitor G protein signaling cycle. Activation of the D2R causes the G protein heterotrimer to dissociate into Gα and Gβγ subunits. Released Gβγ subunits tagged with Venus fluorescent protein interacts with Nluc–tagged reporter G protein receptor kinase (GRK) to produce the BRET signal. Upon termination of D2R activation by antagonist haloperidol, Gαo subunit hydrolyses GTP and reassociates with Gβγ subunits, quenching the BRET signal. (**e**) Time course of normalized BRET responses recorded in a representative experiment. *Left*. The deactivation phase after antagonist application is shown. Wild-type RGS7 or mutant were transfected at equal amount of cDNA (210 ng) together with dopamine D2 receptor, Gαo, and BRET sensor pair. *Right*. Quantification of the exponential decay kinetics of the response. BRET values were averaged from four or six replicates. *P < 0.0001. (**f**) Correlation analysis between expression levels of RGS7 and activity. Expression levels of RGS7 (x axis) were determined by Western blotting (Supplementary Fig. 9c) and plotted against *k*_GAP_ (y axis). Mean ± SEM were shown.

Alongside these known drivers, we identified several novel genes harboring recurrent mutations, one of which was Regulator of G-protein Signaling 7 (*RGS7*). Examination of publically available databases revealed that *RGS7* is also mutated in several other tumor types (Supplementary Fig. 1a,b). Our analysis detected 67 non-synonymous *RGS7* mutations in melanoma samples, 65% of which were predicted by SIFT analysis to be deleterious (Supplementary Table 3). The distribution of protein alterations encoded by the non-synonymous mutations identified in *RGS7* is shown in Fig. 1a. We tested the expression of RGS7 in normal human adult melanocytes and melanoma cells. As seen in Supplementary Fig. 2a, both cell types express RGS7 to various degrees. This result is consistent with previous findings showing that RGS7 is expressed in melanoma^13,14^ and is also indicated in multiple datasets in BioGPS^15^, in cbioportal (Supplementary Table 4) as well as an additional 29 melanoma samples used in this study (Supplementary Fig. 3). To validate the extent to which RGS7 is expressed in human melanomas, we performed RGS7 immunohistochemistry (IHC) on a set of melanoma patient tissues. We found a low/negative expression of RGS7 in 76.2% (48/63) of the cases and moderate expression in the remaining 23.8% (15/63) (Supplementary Fig. 2b). The mutation data for the main melanoma drivers of these samples is provided in Supplementary Table 5.

The most highly recurring mutation in *RGS7* (identified in 11 samples, accounting for ~16% of all RGS7 mutations) was a heterozygous cytosine-to-thymine change at position 130 of the transcript (uc001hyv.2), leading to the substitution of arginine 44 with a cysteine (p.R44C) within the DEP domain of the protein. Importantly, the DEP domain has been shown to play a role in recruiting RGS7 to the plasma membrane, thereby increasing its catalytic activity^16–18^. The p.R44C mutation has also been documented in cancerous samples from hematopoietic and lymphoid tumors and upper aero digestive tract tumors (COSMIC). While the probability of this alteration occurring in melanoma is significantly low (2.7e-14; binomial distribution followed by a Bonferroni correction), the affected residue is highly conserved across species (Supplementary Fig. 4a) and the p.R44C mutation is predicted by SIFT analysis to be deleterious.

Another two mutations found within the protein’s RGS domain were p.E383K and p.R416Q, occurring in six samples and four samples, respectively. The p.E383K mutation comprised a guanine-to-adenine switch at position 1477 of the transcript (uc001hyv.2), leading to substitution of a glutamic acid with a lysine residue; the probability for this occurrence is significantly low (1.45e-05; binomial distribution followed by a Bonferroni correction). The affected residue is conserved across almost all species (Supplementary Fig. 4b) and the p.E383K mutation is predicted by SIFT analysis to be deleterious. The p.R416Q mutant exhibited a guanine-to-adenine change at position 1247 of the transcript (uc001hyv.2), leading to a arginine-to-glutamine substitution. The occurrence probability of this recurrent alteration in melanoma is also low (0.01; binomial distribution followed by a Bonferroni correction*)*. The affected residue is highly conserved across species (Supplementary Fig. 4c), but the p.R416Q mutation is predicted by SIFT analysis to be neutral and was, therefore, not analyzed further in this study.

The evidence described above suggests that mutations at sites 44 and 383 are selected for during tumor development. We, therefore, experimentally tested the hypothesis that these recurrent mutations have a functional role in tumorigenesis. To gain insight into the structural and functional effects of these two deleterious recurrent mutations, we analyzed the 3D structure of the RGS7 protein, focusing on the DEP and RGS domains, where residues 44 and 383 are located, respectively. The structure of the RGS domain of RGS7 (PDB id 2D9J) revealed that residue E383 is likely to establish a salt bridge with R380. The substitution of the negatively charged glutamate with a positively charged lysine in the E383K mutant is predicted to disrupt this interaction (Supplementary Fig. 5). Since the RGS7 DEP domain structure was not determined by crystallography, we generated several model structures based on the highly similar RGS9 structure. The modeling predicted that R44 forms H-bonds with the amino acids in positions 58 and 61 with an R44-S58 distance of 2.1 Å, R44-D61 of 2.8 Å, and E383-R380 of 2.5 Å (Fig. 1b and Supplementary Fig. 6). The R44C mutation is likely to adversely affect protein stability by breaking this conserved H-bond network, thus leading to decreased RGS activity. Interestingly, a mutation at position S58, predicted by SIFT analysis to be deleterious, was also identified in the 501 melanoma samples analyzed. Furthermore, the D61 residue is also highly conserved in the DEP domain across species (Supplementary Fig. 7). Thus, the R44C mutation is likely to affect RGS7 stability by breaking the H-bond network. Indeed, when evaluating protein stability using pulse-chase experiments, we found the stability of R44C to be significantly diminished as compared to wild-type RGS7, supporting the model’s prediction. Importantly, evaluation of E383K stability showed that it is also significantly lower than that of wild-type RGS7 (Fig. 1c and Supplementary Fig. 8).

RGS7 inhibits signaling from a number of Gα_i/o_-coupled GPCRs by virtue of its GAP activity. To test the functional effects of the two recurrent mutations that affect RGS7 stability (R44C and E383K), we studied the activity of wild-type and mutant RGS7 in a GPCR-based system by monitoring real time changes in G protein subunit rearrangement by bioluminescence resonance energy transfer (BRET). The experimental readout quantified the deactivation kinetics of RGS7′s preferred substrate, Gα_o_. According to currently accepted models, constitutively active Gα_o_ is oncogenic^19,20^ and the speed of Gα deactivation is directly proportional to the catalytic activity of the RGS^21^. Thus, the degree by which introduction of RGS7 accelerates Gα_o_ deactivation provides a measure of its function, as the Gα_o_ subunit hydrolyses GTP and re-associates with Gβγ subunits, quenching the BRET signal (Fig. 1d and Supplementary Fig. 9a,b). Consistent with this, we found that wild-type RGS7, as well as E383K, substantially accelerate Gα_o_ deactivation by approximately 3.5 fold (From 0.109 ± 0.003 s^−1^ to 0.382 ± 0.005 s^−1^). In contrast, the R44C mutant was about a third as effective in accelerating Gα_o_ deactivation.

Since the ability of RGS7 to decrease G-protein signaling shows a linear dependence on its concentration^22^, and because the R44C mutation reduces RGS7 stability, we sought to determine whether the reduced activity of the mutant is entirely explained by its lower expression. To address this, we referenced both the catalytic activity and protein levels of the R44C mutant to a calibration plot of respective values for wild-type RGS7. Our results show that, even when normalized for the differences in expression levels, mutant RGS7 still has significantly lower activity than that of the wild-type protein (Fig. 1d,e,f and Supplementary Fig. 9b,c,d), indicating a deficit in the catalytic activity of the mutant protein (p.R44C) in addition to its reduced stability. As RGS7 acts enzymatically, its lower expression results in a corresponding decrease in GPCR regulation (as modeled by the wild-type calibration curve in Fig. 1f). However, the decrease in activity further contributes to the loss of function, and we think that both these changes ultimately produce the loss of function phenotype.

**Figure 2:**
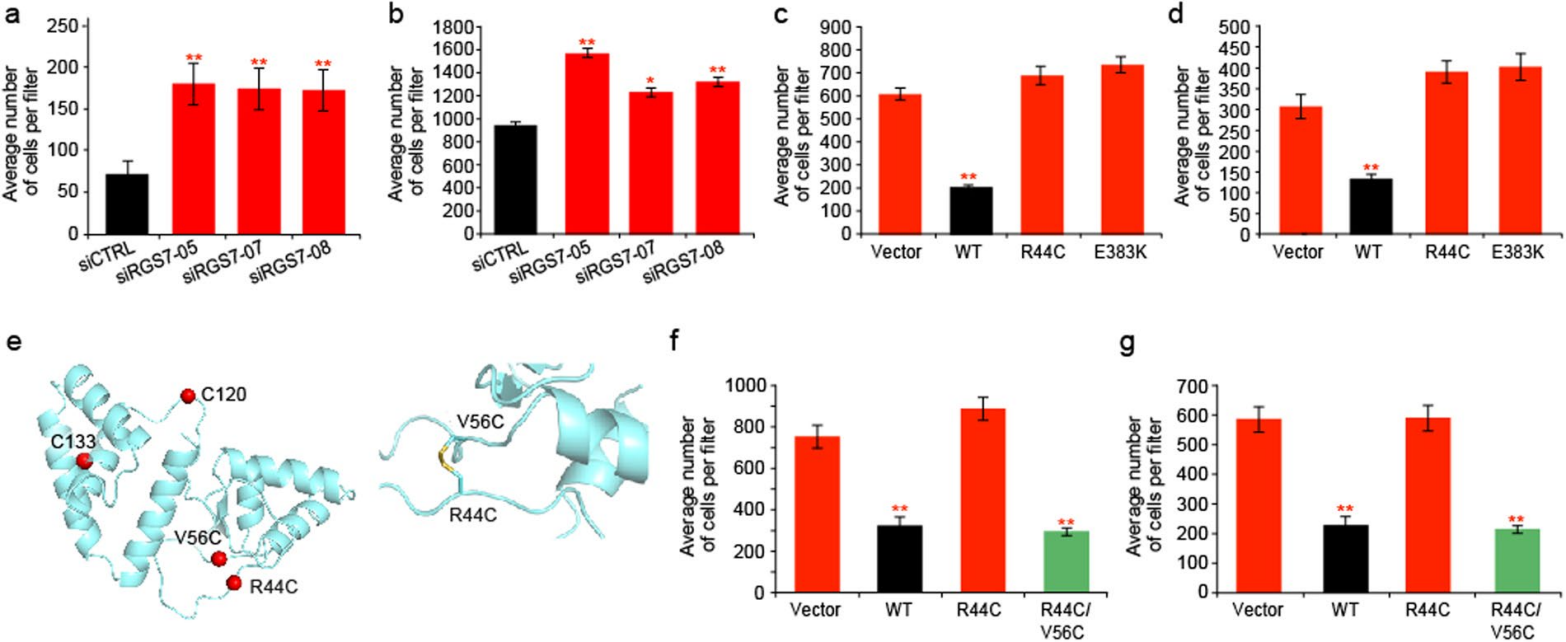
Effects of RGS7 mutations on melanoma cell migration and invasion. (**a**,**b**) A375 cells were depleted for RGS7 using three individual small interfering RNAs (siRNAs) targeting human *RGS7*. A375 was transiently transfected with the indicated vectors for 72 hr. Clones expressing the indicated vectors were seeded in blind well chemotaxis chambers and assessed 16 hr later for their ability to migrate and invade respectively. Stained filters were analyzed using a Nikon Eclipse TS100 microscope 4 × lens and counted with ImageJ software. Quantification made from 2 independent experiments, each done in triplicates. **p < 0.005, *p < 0.05 for wild-type RGS7 versus vector (student’s *t* tests); Error bars, S.D.; WT, wild-type. (**c**,**d**) The migration and invasion ability of A375 expressing wild-type and mutant RGS7 assessed as indicated above. (e-left) The location of cysteine residues in the RGS7 DEP domain, shown as red spheres: C120 and C133 are WT, R44C is the recurrent mutant and V56C is the compensatory point mutation. (e-right) Zoom-in on the disulfide bridge between R44C and V56C. (**f**,**g**) The migration and invasion ability of A375 expressing wild-type, mutant R44C and R44C/V56C assessed as indicated above.

Given that previous studies reported that reduced GAP activity on Gαo increases cell migration and invasion^19,23–26^, we hypothesized that RGS7 functions as a tumor suppressor. Our prediction was confirmed by the finding that knockdown of RGS7, using a pool of siRNAs, significantly increases the migration and invasion of A375 and colo829 melanoma cell lines (P < 0.005, *t* test; Supplementary Fig. 11a,b,c,d). Specific targeting of *RGS7* was confirmed by immunoblotting (Supplementary Fig. 10). To confirm the specificity of the siRNA pool, we used three individual siRNAs to knockdown RGS7 protein levels. We confirmed specific targeting of RGS7 by transfection of A375 and colo829 and immunoblotting for RGS7 (Supplementary Fig. 12). Here, too, depletion of RGS7 with each of the three siRNAs in A375 and colo829 cells significantly increased the cells’ ability to migrate and invade as compared to A375 and colo829 cells targeted with control siRNA (P < 0.005, P < 0.05, *t* test; Fig. 2a,b, Supplementary Fig. 13a,b).

Short hairpin RNA (shRNA) and siRNA-mediated phenotypes could be caused by specific or non-specific effects. We, therefore used a third approach to test the knockdown phenotype. We engineered a 3′ UTR-targeting shRNA construct that cannot target overexpressed RGS7 (siRNA#1), since the exogenous ORF transcript lacks the 3′-UTR sequence targeted by the 3′ UTR-targeting shRNA^27,28^, to rescue the effects of knockdown of endogenous RGS7. A375 and colo829 over-expressing wild-type RGS7 were stably infected either with the non-targeting shRNA construct or with two independent *RGS7*-specific shRNA constructs; an empty vector served as control. We demonstrated that non-targeting shRNA do not knock down RGS7 by transiently transfecting HEK293T cells and immunoblotting for FLAG-RGS7 and GAPDH as a loading control (Supplementary Fig. 14). We further showed by immunoblotting that A375 and colo828 cells expressing the non-targeting shRNA express RGS7 and that the two independent *RGS7*-specific shRNA constructs do knock down RGS7 (Supplementary Fig. 14). Importantly, non-targeting RGS7 shRNA (#1)-reconstituted cells showed significantly less migration and invasion activity than cells infected with shRNAs that do target RGS7 (P < 0.005, P < 0.05, *t* test; Supplementary Fig. 15). These results suggest that RGS7 has a tumor suppressive role in cell migration and invasion in melanoma cells.

We further reasoned that the reduced catalytic activity and stability of R44C, as well as the reduced stability of E383K seen in Fig. 1c,d and Supplementary Figs 8, 9, may also increase basal G protein signaling, thus promoting melanoma cell growth and migration. The activity of RGS7 would, therefore, be required to suppress cellular growth, migration and invasion. To test this possibility, we established stable pooled clones expressing either wild-type RGS7, the R44C mutant, the E383K mutant, or a vector control. All studies were performed in melanoma cell lines A375 and colo829 (Supplementary Fig. 16).

First we examined the effect of RGS7 on cell growth rate. When cultivated on plastic, all clones grew similarly (Supplementary Fig. 17). However, a difference in cell growth was observed when we assessed the cells for anchorage independence, where expression of wild-type but not mutant RGS7 substantially inhibited colony growth on soft agar (P < 0.05, *t*. test; Supplementary Fig. 18).

We also found RGS7 to alter melanoma cell migration and invasion capabilities. Seeded A375 or colo829 pooled clones overexpressing wild-type RGS7 showed lower migration and invasion capacities than those overexpressing mutant RGS7 (P < 0.005, *t* test; Fig. 2c,d and Supplementary Fig. 19). In agreement with the tumor-suppressor role of RGS7, overexpression of wild-type RGS7 in melanoma cell lines harboring the *RGS7* mutation (R44C: 53 T and 67 T) led to reduced cell migration and invasion (Supplementary Figs 20, 21, 22).

As our data indicate that the R44C mutation affects RGS7 stability via disruption of the H-bond network in the DEP domain of RGS7, we were interested to explore the possibility of rescuing the R44C phenotype by designing a compensatory point mutation in the R44C mutant that stabilizes the RGS7 N-terminal domain (Fig. 2e). Among all residues surrounding position 44, we opted to mutate V56 to a cysteine, since the location of V56 allows formation of a disulfide bridge with R44C with minimal disruption to the neighboring structure.

In order to test whether the V56C in RGS7 R44C mutant indeed leads to the formation of a disulfide bond, we overexpressed either WT RGS7, R44C RGS7 or the R44C/V56C mutant RGS7 in A375 cells. We then immunoprecipitated the expressed proteins with a Flag antibody, eluted RGS7 from the beads and the cysteines were modified with N-Ethylmaleimide (NEM) without prior reduction. The samples were then analyzed by LC-MS/MS. Although the expected cross-linked peptide was not identified, a mass corresponding to its cleaved variant was detected. The mass that was observed only in RGS7 R44C/ V56C and not in WT or R44C RGS7 corresponded to: NGIPICTVKSFLSKIPSC (Supplementary Fig. 23). As expected, the introduction of this compensatory point mutation had only a minor effect on RGS7′s catalytic activity (Supplementary Fig. 24a,b). However, it clearly increased RGS7 expression levels, suggesting that the double mutant is, indeed, more stable and that this was achieved by successfully introducing a compensatory mutation that enabled the formation of a new disulfide bridge (Supplementary Fig. 25) without adversely affecting the conformation of the adjacent loop (Supplementary Fig. 26). The rescue mutation reinstated RGS7′s ability to suppress cell migration and invasion, rescuing the deficits observed with the R44C mutant (P < 0.005, *t* test; Fig. 2f,g and Supplementary Fig. 27a,b).

Our discovery of frequent recurrent somatic mutations in RGS7 in melanoma, together with our functional data characterizing the effects of the recurrent mutations (p.R44C and p.E383K) on anchorage-independence, migration and invasion, reveals RGS7 to be an important driver in human melanoma. Similarly, RGS7 expression was reported to be downregulated in high-grade human breast tumors, indicating that this protein may also play a role in breast carcinogenesis^23^. Furthermore, other RGS proteins, such as its close homolog RGS6, have been found to confer inhibitory effects of cancer cell growth^23,29^. Our results are consistent with the canonical function of RGS proteins as key negative regulators of GPCRs, and as pivotal players in cancer development and metastasis^24^. Here, we provide evidence that the RGS7 hotspot mutation at position R44C reduces its ability to inactivate Gα_o_ by breaking a conserved H-bond network with residues S58 and D61, thus destabilizing the protein. The reduced activity of the R44C mutant promotes melanoma cell migration and invasion, providing a molecular mechanism by which RGS7 suppresses melanoma tumorigenesis.

We further show that by engineering the formation of a disulfide bridge between R44C and V56C, the N-terminus of the protein can be re-stabilized and, thereby, its tumor suppressor function rescued. This is particularly interesting, as several RGS proteins have reduced expression or function in pathophysiological states. Our findings suggest that strategies for increasing RGS stability may enhance its function and, therefore, may be clinically relevant.

## Material and Methods

### Tumor tissues

All DNA samples used in this study were derived from melanoma metastases. Samples used for whole-exome capture were extracted from cell lines established directly from patient tumors, as described previously^30^. DNA subjected to whole-genome sequencing was extracted from OCT embedded specimens, as described previously^30^. Tissue was further collected and cell lines established at QIMR Berghofer Medical Research Institute. All cell lines were established as described previously^31^, under a protocol approved by the QIMR Berghofer Medical Research Institute Human Research Ethics Committee, with informed patient consent. Cell line genotypes are provided in Supplementary Table 6. All cell lines have been tested negative for mycoplasma.

All experiments were approved by the Weizmann Institute IRB committee and performed in accordance with relevant guidelines and regulations.

### PCR, sequencing and mutational analysis

PCR and sequencing of *RGS7* were carried out as previously described^32^. Sequence traces were analyzed using the Mutation Surveyor software package (SoftGenetics). Primers used are listed in Supplementary Table 7.

### Antibodies and genetic constructs

Anti-FLAG (M2) (Sigma-Aldrich) (cat no. F7425), anti-GAPDH (Millipore) (cat no. MAB374), anti-cyclin D1 (Cell Signaling) (cat no. 2926) PfuUltra II Hotstart PCR Master Mix (Agilent Technologies, Santa Clara, CA), the pCDF1-MCS2-EF1-Puro vector (Systems Biosciences, Inc., Mountain View, CA), Lipofectamine 2000 (Life Technologies) and cycloheximide (Sigma-Aldrich) were used. Rabbit anti-Gβ5 and rabbit anti-RGS7 7RC-1 were generous gifts from Dr William Simonds^33–44^ and rabbit anti-RGS7 NT was from Dr. Kirill Martemyanov. VSV-G and pFIV-34N were kind gifts from Todd Waldman, Georgetown University. siRNA and shRNA expression constructs were obtained from Open Biosystems.

### Tissue microarray construction

Archival pathology melanoma blocks were available from the Department of Tissue Pathology and Diagnostic Oncology at the Royal Prince Alfred Hospital, Sydney, Australia. A total of 63 archival tissue blocks from melanoma patient specimens were available and used to construct a tissue microarray (TMA). Areas of tumor were identified and a 1.0 mm core was arrayed from each archival tissue block sample.

### Immunohistochemistry for RGS7

Tissue microarray sections were cut at 3 µm onto superfrost + glass slides and stored at 4 °C until IHC was performed (<2 weeks). Immunohistochemistry (IHC) was performed on a Dako autostainer/PT-Link with high pH target retrieval buffer (Dako, K8005) as per manufacturer’s instructions. The primary antibody against RGS7 (RGS7-NT^45^) was incubated for 45 minutes at room temperature at a 1:200 dilution and visualized using the MACH3 Rabbit HRP polymer detection system (Biocare; M3R531) and DAB chromogen kit (Biocare; BDB2004) as per the manufacturer’s instructions.

### Pathological assessment of RGS7 immunohistochemistry staining

A total of 62 tissue array cores were evaluable for RGS7 immunohistochemistry. The predominant RGS7 IHC signal was cytoplasmic and associated with weak membrane staining. RGS7 was evaluated using the intensity of cytoplasmic and membrane tumor signal from 0 to 2 (negative, weak, or moderate).

### Microarray processing

Samples were prepared according to Affymetrix protocols (Affymetrix, Inc). RNA quality and quantity was ensured using the Bioanalyzer (Agilent, Inc) and NanoDrop (Thermo Scientific, Inc) respectively. Per RNA labeling, 300 nanograms of total RNA was used in conjunction with the Affymetrix recommended protocol for the Human Exon 1.0 chips.

The hybridization cocktail containing the fragmented and labeled cDNAs were hybridized to The Affymetrix Human GeneChip® Exon chips. The chips were washed and stained by the Affymetrix Fluidics Station using the standard format and protocols as described by Affymetrix. The probe arrays were stained with streptavidin phycoerythrin solution (Molecular Probes, Carlsbad, CA) and enhanced by using an antibody solution containing 0.5 mg/mL of biotinylated anti-streptavidin (Vector Laboratories, Burlingame, CA). An Affymetrix Gene Chip Scanner 3000 was used to scan the probe arrays. Gene expression intensities were calculated using GeneChip® Command Console® Software (AGCC) and Expression Console™ Software. Cel files generated by the Affymetrix AGCC program were imported in the Partek Genomic Suite software and RMA (Robust Multichip Analysis) normalization, log2 transformation and probe summarization was performed. Anova comparisons and PCA (Principle Component Analysis) were performed within Partek Genomic Suite. RGS7 mRNA expression was evaluated as follows: Relative log2 gene expression values Dot-plot distribution of the RGS7 gene in 30 melanoma tumors and 6 primary melanocytes. The expression value of the whole data set range between 3 and 15. The data was RMA (Robust Multichip Analysis) normalized and a Oneway anova was performed. P-value = 0.212 and 1.18 folds upregulation in the tumors.

### Molecular modeling the three-dimensional (3D) structures of RGS7 wild-type and mutants

The atomic coordinates of RGS7 RGS domain (PDB id 2D9J) were recovered from the Protein Data Bank (http://www.pdb.org)^46^. E383K mutant was manually created and then was minimized using the Polak-Ribiere Conjugate Gradient (PRCG) minimization algorithm, the OPLS2005 force field and implicit solvent available in MacroModel v. 10.8 (Schrödinger, LLC, New York, NY, 2015).

RGS7 N-terminal domain was modeled using the full-length X-ray structure of the homologous RGS9 (in complex with Gβ5, PDB id 2PBI)^47^, which has 40% identity and 56% similarity to RGS7). Chain A was selected as template for modeling automatically by I-TASSER v. 4.4^48^ and the Phyre2^49^ web servers, and manually by Modeller v. 9.15^50^ and Prime v. 4.0 (Schrödinger, LLC, New York, NY, 2015)^51,52^. Due to the high sequence similarity between the RGS7 and RGS9 N-terminal domains, the same sequence alignment was obtained by different methods. Using default settings, five models were generated by I-TASSER and Modeller, and one model by Prime and Phyre2, respectively. All models were used as input for a side chain refinement with SCWRL4^53^. After side chain refinement, we observed that orientations of the R44 residue in the different models cluster to similar positions (Supplementary Fig. 6). R44 switches between alternative conformations where it establishes H-bonds with S58, with D61, or with both S58 and D61. The representative structure is shown in Fig. 1b. Residues around R44C were analyzed as potential positions for cysteine substitutions to form a disulfide bridge (Supplementary Fig. 26). H-bonds are automatically visualized according to default distance criteria defined in the software Maestro (Schrodinger): maximum distance of 2.8 Å, donor minimum angle of 120, acceptor minimum angle of 90. The distances are calculated between the hydrogen and acceptor atom, and can be manually measured by the “Measure” tool available in Maestro.

The substitution of an arginine with the shorter cysteine in the mutant RGS7 makes positions 58 and 61 too far from 44 to allow for disulfide bridge formation without affecting the loop conformation. In contrast, V56C resulted in optimal distance for a disulfide bond formation with R44C (Cα^56^-Cα^44^ distance of 5.3 Å, Cβ^56^-Cβ^44^ distance of 4.0 Å)^54^. The V56C and R44C mutations and the disulfide bridge were manually created in the representative model of the wild-type RGS7 model. The resulting R44C/V56C model was minimized using the Polak-Ribiere Conjugate Gradient (PRCG) minimization algorithm, the OPLS2005 force field and implicit solvent available in MacroModel v. 10.8 (Schrödinger, LLC, New York, NY, 2015). Native cysteines in this domain are located too far to be able to form alternative disulfide bridges (Fig. 2e).

### Pulse chase experiments for RGS7 stability determination

HEK293T Cells (3.0** × **10^6^) were seeded in 10 cm dishes 24 hr before transfection. Cells were transfected with *RGS7* (wild-type, R44C, E383K or R44C/V65C) and *GB5* at a 1:1 ratio. 24 h later, cells were sub-cultured into 6-well dishes (7 × 10^5^ cells per well). The next day, cells were treated with protein synthesis inhibitor cycloheximide (40 μg/ml) and collected at different time points. The amount of *RGS7* was determined by Western blot. Cyclin D1 served as a control.

### Monitoring G protein cycle in live cells by BRET assay

BRET experiments were performed as previously reported with slight modifications^55,56^. Briefly, HEK293T/17 cells were grown in DMEM supplemented with 10% FBS, minimum Eagle’s medium non-essential amino acids, 1 mm sodium pyruvate, and antibiotics (100 units/ml penicillin and 100 μg/ml streptomycin) at 37 °C in a humidified incubator containing 5% CO_2_. Cells were transfected with PLUS (7.5μl per 6-cm dish) and Lipofectamine LTX (12 μl per 6-cm dish) reagents. BRET measurements were made using a microplate reader (POLARstar Omega, BMG Labtech) equipped with two emission photomultiplier tubes. All measurements were performed at room temperature. The BRET signal is determined by calculating the ratio of the light emitted by Gβ1γ2-Venus (535 nm) over the light emitted by masGRK3ct-Nluc (475 nm). The average base-line value recorded prior to agonist stimulation was subtracted from BRET signal values, and the resulting difference (ΔBRET ratio) was normalized to the response immediately before haloperidol application. The rate constants (1/τ) of the deactivation phases were obtained by fitting a single exponential curve to the traces. *k*_GAP_ rate constants were determined by subtracting the basal deactivation rate (*k*_app_) from the deactivation rate measured in the presence of exogenous RGS protein. Obtained *k*_GAP_ rate constants were used to quantify GAP activity.

### Construction of wild-type and mutant expression vectors

Human *RGS7* cDNA (NM_001282778) was cloned from HEK293T cDNA using PfuUltra II Hotstart PCR Master Mix (Agilent Technologies, Santa Clara, CA) according to manufacturers’ instructions, using the following forward and reverse primers: tgaccaggatccgccaccatggcccaggggaataattatgggcagaccagc, ggtcagcggccgctcacttatcgtcgtcatccttgtaatctaacaggttagtgctggccc. A FLAG tag was introduced onto the C-terminus of *RGS7* during the cloning procedure. PCR products were cloned into the pCDF1-MCS2-EF1-Puro vector via the BamHI and NotI restriction sites. The p.R44C mutation was introduced using fusion PCR site-directed mutagenesis, using the following forward and reverse primers: ggaattcctatttgtacggtcaaaagc, gcttttgaccgtacaaataggaattcc. The p.E383K was introduced into the wild-type using Q5 polymerase (NEB), phosphorylating the PCR product using PNK (NEB) and self ligating it using Quick Ligase (NEB). Primer pairs were: gaaaatatggcaagagtttctgg, ctgaactcttgagggtacttctttaata. The p.V56C, point mutation was introduced into the p.R44C by synthesizing the full vector using Q5 polymerase (NEB), phosphorylating the PCR product using PNK (NEB) and self ligating it using Quick Ligase (NEB). Primer pairs were: GCTTCTCTGGTTCAGACATTGTT, AGCTAGGTATCTTGGAAAGAAAGC.

### Establishment of overexpressing cell lines

To produce lentivirus, *RGS7* constructs were co-transfected with pVSV-G and pFIV-34N helper plasmids into HEK 293 T cells seeded at 2. 5 × 10^6^ per T75 flask, using Lipofectamine 2000, per manufacturer’s instructions. Virus-containing media was harvested 60 h after transfection, filtered, aliquoted and stored at −80 °C. A375, colo829, 67 T and 53 T cells were grown in RPMI-1640 (Biological Industries) supplemented with 10% FBS (HyClone, Logan, UT). Lentivirus for *RGS7* (wild-type, R44C and E383K) and empty vector control were used to infect the cells as previously described^57^.

### Transient transfection of wild-type and mutant RGS7

A375 and colo829 Cells (3.0** × **10^6^) were seeded in 10 cm dishes 24 hr before transfection. Cells were transfected with empty vector control and *RGS7* (wild-type, R44C, E383K and R44C/V65C) using TurboFect per manufacturer’s instructions. RGS7 levels were detected by immunoblotting (Supplementary Fig. 24).

### siRNA depletion of endogenous *RGS7*

Smart Pool siRNA specific to human *RGS7* (ON-TARGETplus) (cat no. L-015720-00-0020) as well as single siRNAs (cat no. LU-002000-00-0008) were purchased from Dharmacon. Sequences of the siRNAs used to transiently deplete *RGS7* in melanoma cell lines are provided in Supplementary Table 8. Using DharmaFECT Transfection Reagent 1 (specific for siRNA), melanoma cell lines were transfected with 50 nM ON-TARGET siRNA in OptiMEM-I medium (Life Technologies). Cells were incubated for 72 hr after transfection, before they were tested in functional assays. The targeting of *RGS7* by siRNAs was confirmed to efficiently knockdown RGS7 at the protein level (Supplementary Figs 10, 12).

### Rescue of RGS7 expression and migratory phenotype by an exogenous non-targeting shRNA

Constructs for stable depletion of *RGS7* and a 3′ UTR- shRNA construct that cannot target overexpressed RGS7 (siRNA#1) were obtained from Open Biosystems (cat no. RHS4533-EG6000). Sequences of the shRNAs used are provided in Supplementary Table 9. HEK293T cells were transiently transfected with shRNA constructs and immunoblotted for FLAG-RGS7 and GAPDH as a loading control (Supplementary Fig. 14). Lentiviral stocks were prepared as previously described^32^. A375 and colo829 cells expressing wild-type RGS7 were stably infected with a 3′ UTR-targeting short hairpin RNA (shRNA) construct as well as two independent *RGS7*-specific shRNAs constructs and selected as previously described^32^. Selection of stable pooled clones was done in puromycin-containing normal medium for 3–5 days, before determining knockdown efficiency. The targeting of *RGS7* by the two independent *RGS7*-specific shRNAs constructs to efficiently knockdown RGS7 at the protein level and the rescue of RGS7 expression using a non-targeting shRNA was confirmed by immunoblotting (Supplementary Fig. 14). Stably infected pooled clones were tested in functional assays.

### Soft agar assay

A375 and colo829 pooled clones overexpressing RGS7 were plated in 4 replicates at 1000 cells/well and 2000 cells per well, respectively, in top plugs of a 24-well plate, consisting of sterile 0.33% Bacto-Agar (BD, Sparks, MD) and 10% FBS (HyClone, Logan, UT). The lower plug contained sterile 0.5% Bacto-Agar and 10% FBS. After two weeks, colonies were counted.

### Proliferation assay

To examine cell growth, A375 and colo829 pooled RGS7 overexpressing clones were seeded in six replicates in 96-well plates, at 200–2,000 cells per well, and incubated for 7–17 days. Samples were analyzed every 48 h by lysing cells in 50 μl of 0.2% SDS/well and incubating for 2 h at 37 °C before the addition of 150 μl/well of SYBR Green I solution (1:750 dilution of SYBR Green I (Invitrogen-Molecular Probes) in distilled water).

### Migration and Invasion Assays

Blind well chemotaxis chambers with 13 mm diameter, 8 mm pore size PVPF filters (Costar Scientific Co, Cambridge, MA) were used. In the invasion assays the chambers were coated with matrigel. Cells (3 × 10^5^), suspended in serum free medium, were added to the upper chamber. 10% FBS full medium was placed in the lower chamber. Assays were carried out at 37 ^o^C in 5% CO_2_. After incubation (16 hr), the upper surface of the filter was freed of cells, using a cotton swab. Cells that passed through the filter to the bottom side were fixed in methanol and then stained with Geimsa. Each triplicate assay was performed three times. Migrating cells were counted blindly in ten representative light-microscopy fields.

### Western Blotting

A375 and colo829 cells stably transfected with RGS7-FLAG (wild-type or mutant) and 67 T and 53 T cells stably transfected with RGS7-FLAG (wild-type or empty vector), were gently washed twice in PBS, then lysed using 2 × sample buffer and freshly added 4% β-mercaptoethanol. Lysed cells were scraped, transferred into a 1.5 mL microcentrifuge tube and sonicated. Proteins were resolved in 10% SDS-polyacrylamide gels and transferred to nitrocellulose membranes (BioRad). Western blots were probed with the following antibodies: anti-FLAG (M2)- (Sigma-Aldrich) and anti-GAPDH (Millipore). For Western blotting of the BRET assay, ∼5 × 10^6^ cells were lysed in 500 μl of sample buffer (125 mm Tris (pH 6.8), 4 m urea, 4% SDS, 10% 2-mercaptoethanol, 20% glycerol, 0.16 mg/ml bromphenol blue). The relative expression level of RGS7 was determined by subtracting the background densities in the absence of exogenous RGS7 and normalizing the resulting value as a fraction of the brightest band intensity expressing the maximal amount of RGS7.

### Evaluation of S-S bond formation in RGS7 R44C/ V56C

WT RGS7, R44C RGS7 or R44C/V56C mutant RGS7 were overexpressed in A375  cells, immunoprecipitated with a Flag antibody, eluted from the beads and the cysteine were modified with N-Ethylmaleimide (NEM) without prior reduction in order to block formation of new disulfide bonds. The samples were then separated on SDS-PAGE and the slices at the expected size were cut. The samples were digested by trypsin and analyzed by LC-MS/MS on Q-Exactive-plus mass spectrometer fitted with a capillary HPLC (Thermo-Fisher Scientific). The peptides were identified by Discoverer software version 1.4 vs human uniprot and decoy databases (in order to determine the false discovery rate (FDR), and versus the specific sequences, using the Sequest search engine. Although the MS/MS spectrum was not clear enough to provide a statistically significant identification, the expected mass of the peptides was accurately observed.

### Statistical analyses

We generate p-values (two-tailed t-test) using Microsoft Excel, to determine significance. We considered p-values below 0.005 to be statistically significant. Frameshift, nonsense and deleterious mutations were predicted by SIFT analysis and considered as deleterious. For the analysis of the data reporting the difference in exponential rate constant of the G protein deactivation kinetics observed in BRET assay experiments, we used one-way ANOVA followed by Dunett’s post-hoc test with GraphPad Prism 6. Linear regression was used to relate the *k*_GAP_ value to the expression level of wild-type RGS7.

### Statistical calculation of the likelihood of a recurrent mutation

The probability of a specific base mutated in 11/501 samples is calculated using the binomial distribution, assuming a background mutation rate of 28.8 mut/Mb (dipyrimidine mutation rate) employing the following values and formula: x = 11, n = 501, P = 28.8e−6.

$$(F(x;n,p)={\rm{\Pr }}(X\le x)=\sum _{i=0}^{[x]}{(}_{i}^{n}){p}^{i}{(1-p)}^{n-1}$$

This result is then corrected for multiple comparisons, to arrive at the probability of any base mutated at 11/501 in the study, by using conservative Bonferroni correction, such that the resulting number is multiplied by the total number of sequenced coding bases.

## Electronic supplementary material

Supplementary figures

Supplementary Table 4

Supplementary Table 5

## References

[1] Siegel R, Naishadham D, Jemal A (2012). Cancer statistics, 2012. CA Cancer J Clin.

[2] Chapman PB (2011). Improved survival with vemurafenib in melanoma with BRAF V600E mutation. The New England journal of medicine.

[3] Flaherty KT (2010). Inhibition of mutated, activated BRAF in metastatic melanoma. N Engl J Med.

[4] Davies H (2002). Mutations of the BRAF gene in human cancer. Nature.

[5] Flaherty KT, Hodi FS, Fisher DE (2012). From genes to drugs: targeted strategies for melanoma. Nat Rev Cancer.

[6] Hennessy BT, Smith DL, Ram PT, Lu Y, Mills GB (2005). Exploiting the PI3K/AKT pathway for cancer drug discovery. Nat Rev Drug Discov.

[7] Hamilton AL (2005). Proteasome inhibition with bortezomib (PS-341): a phase I study with pharmacodynamic end points using a day 1 and day 4 schedule in a 14-day cycle. J Clin Oncol.

[8] Herbst RS, Phase I (2002). Study of Recombinant Human Endostatin in Patients With Advanced Solid Tumors. Journal of Clinical Oncology.

[9] Huang S (2003). Targeting mTOR signaling for cancer therapy. Current Opinion in Pharmacology.

[10] Sjoblom T (2006). The consensus coding sequences of human breast and colorectal cancers. Science (New York, N.Y.

[11] Hodis E (2012). A landscape of driver mutations in melanoma. Cell.

[12] Krauthammer M (2012). Exome sequencing identifies recurrent somatic RAC1 mutations in melanoma. Nat Genet.

[13] Xu L (2008). Gene expression changes in an animal melanoma model correlate with aggressiveness of human melanoma metastases. Molecular cancer research: MCR.

[14] Ulloa-Montoya F (2013). Predictive gene signature in MAGE-A3 antigen-specific cancer immunotherapy. Journal of clinical oncology: official journal of the American Society of Clinical Oncology.

[15] Wu C, Jin X, Tsueng G, Afrasiabi C, Su AI (2016). BioGPS: building your own mash-up of gene annotations and expression profiles. Nucleic Acids Res.

[16] Drenan RM (2006). R7BP augments the function of RGS7*Gbeta5 complexes by a plasma membrane-targeting mechanism. The Journal of biological chemistry.

[17] Kirill A, Martemyanov (2003). TheDEP Domain Determines Subcellular Targeting of the GTPase Activating Protein RGS9 *In Vivo*. The Journal of Neuroscience.

[18] Martemyanov KA, Yoo PJ, Skiba NP, Arshavsky VY (2005). R7BP, a Novel Neuronal Protein Interacting with RGS Proteins of the R7 Family. Journal of Biological Chemistry.

[19] R PT, Horvath CM, R. I (2000). Stat3-mediated transformation of NIH-3T3 cells by the constitutively active Q205L Galphao protein. Science.

[20] Krolls SD (1992). The Q205LGo-a! Subunit Expressed in NIH-3T3 Cells Induces Transformation*. THEJ OURNALO F BIOLOGICACLH EMISTRY.

[21] Muntean BS, Martemyanov KA (2016). Association with the Plasma Membrane Is Sufficient for Potentiating Catalytic Activity of Regulators of G Protein Signaling (RGS) Proteins of the R7 Subfamily. The Journal of biological chemistry.

[22] Masuho I, Xie K, Martemyanov KA (2013). Macromolecular Composition Dictates Receptor and G Protein Selectivity of Regulator of G Protein Signaling (RGS) 7 and 9-2 Protein Complexes in Living Cells. The Journal of biological chemistry.

[23] Maity B (2013). Regulator of G protein signaling 6 is a novel suppressor of breast tumor initiation and progression. Carcinogenesis.

[24] Lappano R, Maggiolini M (2011). G protein-coupled receptors: novel targets for drug discovery in cancer. Nature reviews. Drug discovery.

[25] Xie Y (2009). Breast cancer migration and invasion depend on proteasome degradation of regulator of G-protein signaling 4. Cancer research.

[26] Ram PT, R. G Iyengar1 (2001). protein coupled receptor signaling through the Src and Stat3 pathway: role in proliferation and transformation. Oncogene.

[27] Jackson AL (2003). Expression profiling reveals off-target gene regulation by RNAi. Nature biotechnology.

[28] Lipinski CA, Lombardo F, Dominy BW, Feeney (2001). P. J. Experimental and computational approaches to estimate solubility and permeability in drug discovery and developmentq settings. Adv Drug Deliv Rev..

[29] Huang, J., Yang, J., Maity, B., Mayuzumi, D. & Fisher, R. Regulator of G protein signaling 6 mediates doxorubicin-induced ATM and p53 activation by a reactive oxygen species-dependent mechanism. *Cancer Res*. **71** (2011). 10.1158/0008-5472.CAN-10-3397 PMC3196377 21859827

[30] Wei X (2011). Exome sequencing identifies GRIN2A as frequently mutated in melanoma. Nat Genet.

[31] Dutton-Regester K (2014). A highly recurrent RPS27 5′UTR mutation in melanoma. Oncotarget.

[32] Palavalli LH (2009). Analysis of the matrix metalloproteinase family reveals that MMP8 is often mutated in melanoma. Nat Genet.

[33] Orlandi C (2015). Orphan Receptor GPR158 Is an Allosteric Modulator of RGS7 Catalytic Activity with an Essential Role in Dictating Its Expression and Localization in the Brain. The Journal of biological chemistry.

[34] Orlandi C (2012). GPR158/179 regulate G protein signaling by controlling localization and activity of the RGS7 complexes. The Journal of cell biology.

[35] Nini L, Zhang JH, Pandey M, Panicker LM, Simonds WF (2012). Expression of the Gbeta5/R7-RGS protein complex in pituitary and pancreatic islet cells. Endocrine.

[36] Cao Y (2012). Regulators of G protein signaling RGS7 and RGS11 determine the onset of the light response in ON bipolar neurons. Proceedings of the National Academy of Sciences of the United States of America.

[37] Panicker LM (2010). Nuclear localization of the G protein β5/R7-regulator of G protein signaling protein complex is dependent on R7 binding protein. Journal of Neurochemistry.

[38] Chen FS (2010). Functional redundancy of R7 RGS proteins in ON*-*bipolar cell dendrites. Investigative ophthalmology & visual science.

[39] Anderson GR, Lujan R, Martemyanov KA (2009). Changes in striatal signaling induce remodeling of RGS complexes containing Gbeta5 and R7BP subunits. Molecular and cellular biology.

[40] Cao Y (2009). Retina-specific GTPase accelerator RGS11/G beta 5S/R9AP is a constitutive heterotrimer selectively targeted to mGluR6 in ON-bipolar neurons. The Journal of neuroscience: the official journal of the Society for Neuroscience.

[41] Cao Y (2008). Targeting of RGS7/Gbeta5 to the dendritic tips of ON-bipolar cells is independent of its association with membrane anchor R7BP. The Journal of neuroscience: the official journal of the Society for Neuroscience.

[42] Anderson GR, Semenov A, Song JH, Martemyanov KA (2007). The membrane anchor R7BP controls the proteolytic stability of the striatal specific RGS protein, RGS9-2. The Journal of biological chemistry.

[43] Nini L (2007). R7-binding protein targets the G protein β5/R7-regulator of G protein signaling complex to lipid rafts in neuronal cells and brain. BMC Biochemistry.

[44] Rojkova AM (2003). Ggamma subunit-selective G protein beta 5 mutant defines regulators of G protein signaling protein binding requirement for nuclear localization. The Journal of biological chemistry.

[45] Aguado, C. *et al*. Cellular and Subcellular Localization of the RGS7/Gβ5/R7BP Complex in the CerebellarCortex. *Frontiers in Neuroanatomy* 1**0**, 10.3389/fnana.2016.00114 (2016). 10.3389/fnana.2016.00114 PMC5127842 27965545

[46] Helen M (2000). Berman *et al*. The Protein Data Bank. Nucleic Acids Res.

[47] Cheever ML (2008). Crystal structure of the multifunctional Gbeta5-RGS9 complex. Nat Struct Mol Biol.

[48] Yang J, Zhang Y (2015). I-TASSER server: new development for protein structure and function predictions. Nucleic Acids Res.

[49] Kelley LA, Mezulis S, Yates CM, Wass MN, Sternberg MJE (2015). The Phyre2 web portal for protein modeling, prediction and analysis. Nat Protoc.

[50] Webb B, Sali A (2014). Protein structure modeling with MODELLER. Methods Mol Biol.

[51] Jacobson MP (2004). A hierarchical approach to all-atom protein loop prediction. Proteins.

[52] Jacobson MP, Friesner RA, Xiang Z, Honig B (2002). On the Role of the Crystal Environment in Determining Protein Side-chain Conformations. Journal of Molecular Biology.

[53] Krivov GG, Shapovalov MV, Dunbrack RL (2009). Improved prediction of protein side-chain conformations with SCWRL4. Proteins.

[54] Sowdhamini R (1989). Stereochemical modeling of disulfide bridges. Criteria for introduction into proteins by site-directed mutagenesis. Protein Engineering.

[55] Ikuo Masuho *et al*. Distinct profiles of functional discrimination among G proteins determine the actions of G protein–coupled receptors. *Science Signaling* **8**, ra123 (2015). 10.1126/scisignal.aab4068 PMC4886239 26628681

[56] Masuho I, Martemyanov KA, Lambert NA (2015). Monitoring G Protein Activation in Cells with BRET. Methods in molecular biology.

[57] Solomon DA (2008). Mutational inactivation of PTPRD in glioblastoma multiforme and malignant melanoma. Cancer research.

