# Author Correction: RGS7 is recurrently mutated in melanoma and promotes migration and invasion of human cancer cells

**DOI:** 10.1038/s41598-018-37932-6

**Published:** 2019-03-09

**Authors:** Nouar Qutob, Ikuo Masuho, Michal Alon, Rafi Emmanuel, Isadora Cohen, Antonella Di Pizio, Jason Madore, Abdel Elkahloun, Tamar Ziv, Ronen Levy, Jared J. Gartner, Victoria K. Hill, Jimmy C. Lin, Yael Hevroni, Polina Greenberg, Alexandra Brodezki, Steven A. Rosenberg, Mickey Kosloff, Nicholas K. Hayward, Arie Admon, Masha Y. Niv, Richard A. Scolyer, Kirill A. Martemyanov, Yardena Samuels

**Affiliations:** 1 Molecular Cell Biology Department, Weizmann Institute of Science, Rehovot, Israel; 2 Department of Neuroscience, The Scripps Research Institute, FL, 33458 USA; 3 Institute of Biochemistry, Food Science and Nutrition, The Robert H. Smith Faculty of Agriculture, Food, and Environment, The Hebrew University, Rehovot, Israel; 4 Melanoma Institute Australia, University of Sydney, NSW, Australia; 5 Tissue Pathology and Diagnostic Oncology, Royal Prince Alfred Hospital, NSW, Australia; 6 National Human Genome Research Institute, US National Institutes of Health, Bethesda, Maryland USA; 7 Department of Biology, Technion-Israel Institute of Technology, Haifa, Israel; 8 National Cancer Institute, Surgery Branch, US National Institutes of Health, Bethesda, Maryland 20892 USA; 9 Department of Human Biology, Faculty of Natural Sciences, University of Haifa, Haifa, Israel; 10 QIMR Berghofer Medical Research Institute, Brisbane, Queensland Australia; 11 Disciplines of Surgery and Pathology, Sydney Medical School, The University of Sydney, Sydney, NSW Australia

Correction to: *Scientific Reports* 10.1038/s41598-017-18851-4, published online 12 January 2018

The Acknowledgements section in this Article is incomplete.

“We thank Mr. N. K. Skamangas for technical support and Dr. A. Kovoor for the D2 receptor expression plasmid, Dr. H. Itoh for GαoA and Dr. N. Lambert for sharing the Gβγ-Venus constructs. This work was supported by NIH grants DA036596 and DA026405 (KAM). MYN is supported by Israel Science Foundation grant 432/12 and Chief Scientist Ministry of Health (the ERA-NET network) and grant no. 3-9543 from the Chief Scientist Office of the Ministry of Health, Israel via the ERA-net network. MK is supported by Israel Science Foundation grant numbers 1454/13, 1959/13, 2155/15. YS is supported by the Israel Science Foundation grant numbers 1604/13 and 877/13, the ERC (StG-335377), by the Henry Chanoch Krenter Institute for Biomedical Imaging and Genomics, the estate of Alice Schwarz-Gardos, the estate of John Hunter, the Knell Family, the Peter and Patricia Gruber Award and the Hamburger Family. Lady Davis Fellowship to ADP is gratefully acknowledged. NKH and RAS are supported by fellowships from the National Health and Medical Research Council of Australia.”

should read:

“We thank Mr. N. K. Skamangas for technical support and Dr. A. Kovoor for the D2 receptor expression plasmid, Dr. H. Itoh for GαoA and Dr. N. Lambert for sharing the Gβγ-Venus constructs. This work was supported by NIH grants DA036596 and DA026405 (KAM). MYN is supported by Israel Science Foundation grant 432/12 and Chief Scientist Ministry of Health (the ERA-NET network) and grant no. 3-9543 from the Chief Scientist Office of the Ministry of Health, Israel via the ERA-net network. MK is supported by Israel Science Foundation grant numbers 1454/13, 1959/13, 2155/15. YS is supported by the Israel Science Foundation grant numbers 1604/13 and 877/13, the ERC (StG-335377), The Minerva Foundation Grant, by the Henry Chanoch Krenter Institute for Biomedical Imaging and Genomics, the estate of Alice Schwarz-Gardos, the estate of John Hunter, the Knell Family, the Peter and Patricia Gruber Award and the Hamburger Family. Lady Davis Fellowship to ADP is gratefully acknowledged. NKH and RAS are supported by fellowships from the National Health and Medical Research Council of Australia.”

