# Supplementary material for: RGS7 is recurrently mutated in melanoma and promotes migration and invasion of human cancer cells
Source: Sci Rep. 2018 Jan 12;8:653. doi: 10.1038/s41598-017-18851-4 (PMC5766496; doi:10.1038/s41598-017-18851-4)
Supplement: Supplementary file 1 — Supplementary figures [file 41598_2017_18851_MOESM1_ESM.pdf]

Supplementary Figures

RGS7 is recurrently mutated in melanoma and promotes migration and invasion of human cancer cells

Nouar Qutob[1], Ikuo Masuho[2], Michal Alon[1], Rafi Emmanuel[1,] Isadora Cohen[1], Antonella Di Pizio[3], Jason Madore[4,5], Abdel Elkahloun[6], Tamar Ziv[7], Ronen Levy[1], Jared J. Gartner[8], Victoria K. Hill[6], Jimmy C. Lin[6], Yael Hevroni[1], Polina Greenberg[1], Alexandra Brodezki[1], Steven A. Rosenberg[6], Mickey Kosloff[9], Nicholas K. Hayward[4,10], Arie Admon[7], Masha Y. Niv[3], Richard A. Scolyer[4,5,11], Kirill A. Martemyanov[2], Yardena Samuels[1]*

[1] Molecular Cell Biology Department, Weizmann Institute of Science, Rehovot, Israel
[2] Department of Neuroscience, The Scripps Research Institute, FL 33458, USA

[3] Institute of Biochemistry, Food Science and Nutrition, The Robert H Smith Faculty of Agriculture, Food and Environment, The Hebrew University, Israel

[4] Melanoma Institute Australia, University of Sydney, NSW, Australia

[5]Tissue Pathology and Diagnostic Oncology, Royal Prince Alfred Hospital, NSW, Australia

[6] National Human Genome Research Institute, US National Institutes of Health, Bethesda, Maryland, USA

[7]Department of Biology, Technion - Israel Institute of Technology, Haifa, Israel

[8]National Cancer Institute, Surgery Branch, US National Institutes of Health, Bethesda, Maryland, 20892, USA
[9]Department of Human Biology, Faculty of Natural Sciences, University of Haifa, Israel

[10] QIMR Berghofer Medical Research Institute, Brisbane, Queensland, Australia

[11]Disciplines of Surgery and Pathology, Sydney Medical School, The University of Sydney, Sydney, NSW, Australia*To whom correspondence should be addressed:
Email: Yardena.Samuels@weizmann.ac.il

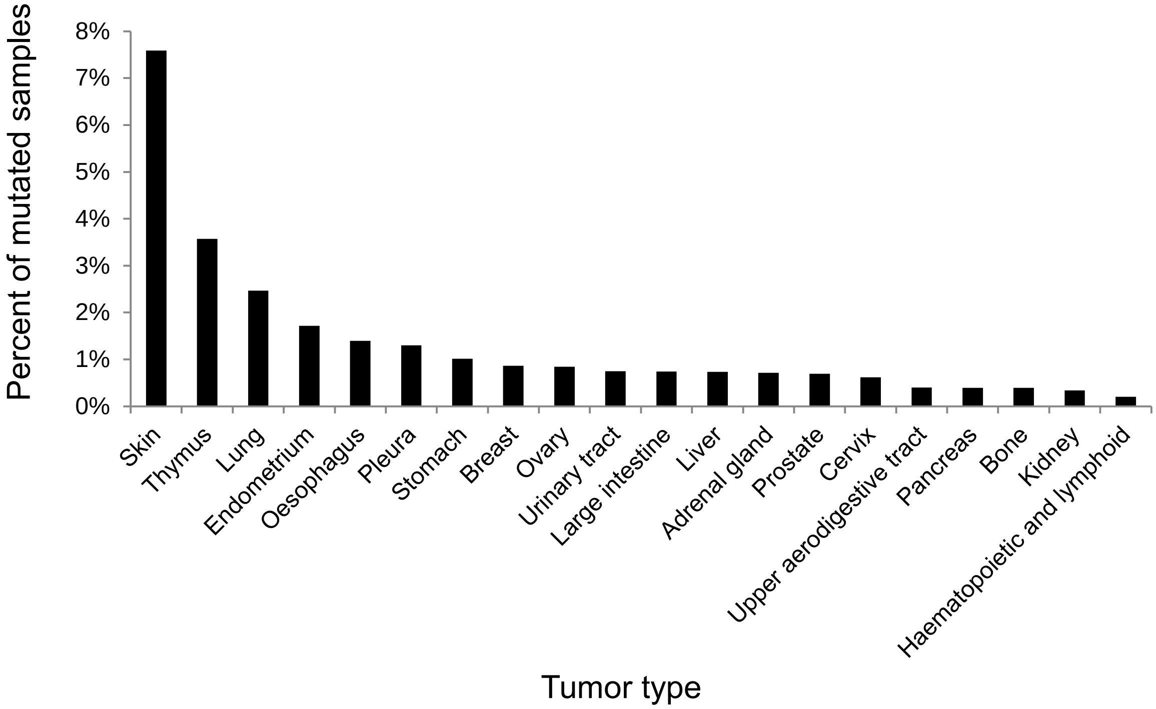

Supplementary Figure 1a. Summary of non-synonymous mutations in *RGS7* across different tumor types from COSMIC.

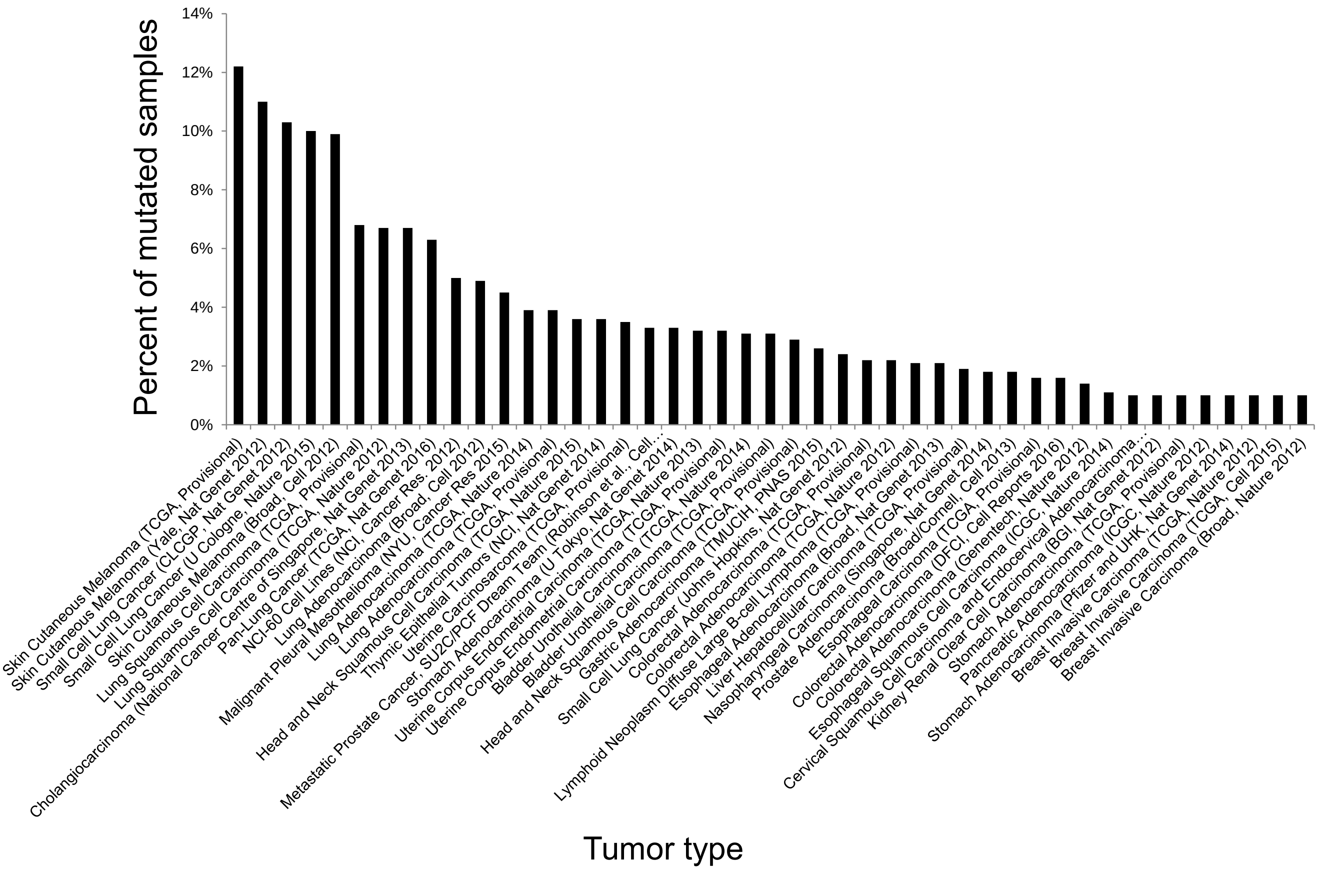

Supplementary Figure 1b. Summary of non-synonymous mutations in *RGS7* across different tumor types from cBioPortal.

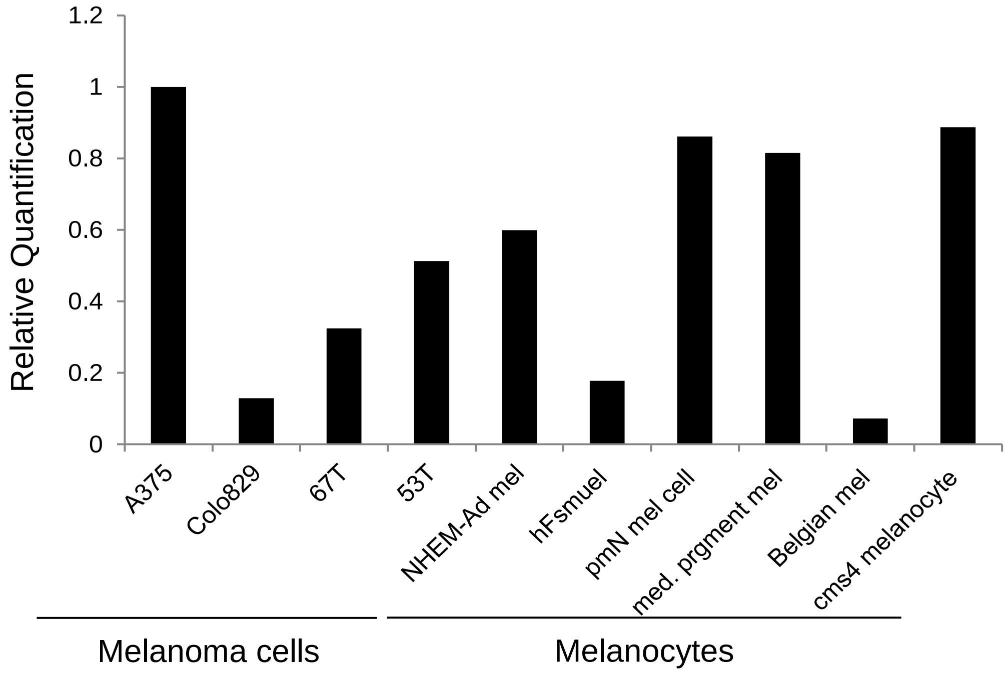

Supplementary Figure 2a. RGS7 mRNA expression in melanocytes and melanoma cells. Detection of the RGS7 mRNA levels in melanoma cells (A375, Colo829, 67T, 53T) and melanocytes (NHEM-Ad mel, hfsmuel, pmN mel cell, med. prgment mel, Belgian mel, cms4 melanocyte) was done by RT-PCR using HPRT as internal control.

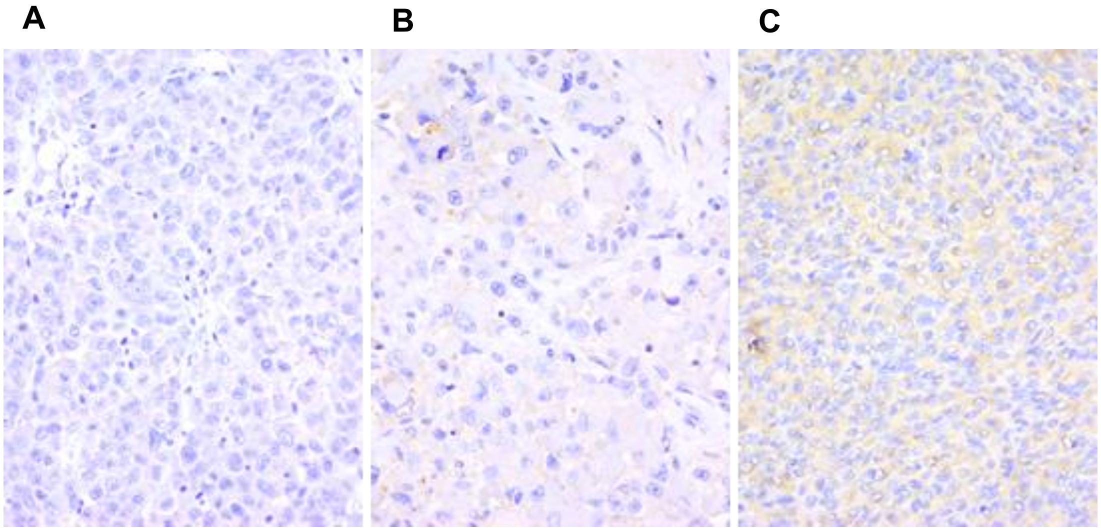

Supplementary Figure 2b. Immunohistochemistry for RGS7 in melanoma tumours. A total of 62 tissue array cores were evaluable for RGS7 immunohistochemistry. The predominant RGS7 IHC signal was cytoplasmic and associated with weak membrane staining. RGS7 was evaluated using the intensity of cytoplasmic and membrane tumor signal from 0 to 2 (negative, weak, or moderate). Sample images of A) Negative RGS7 staining (score 0). B) Weak RGS7 staining (score 1). C) Moderate RGS7 staining (score 2).

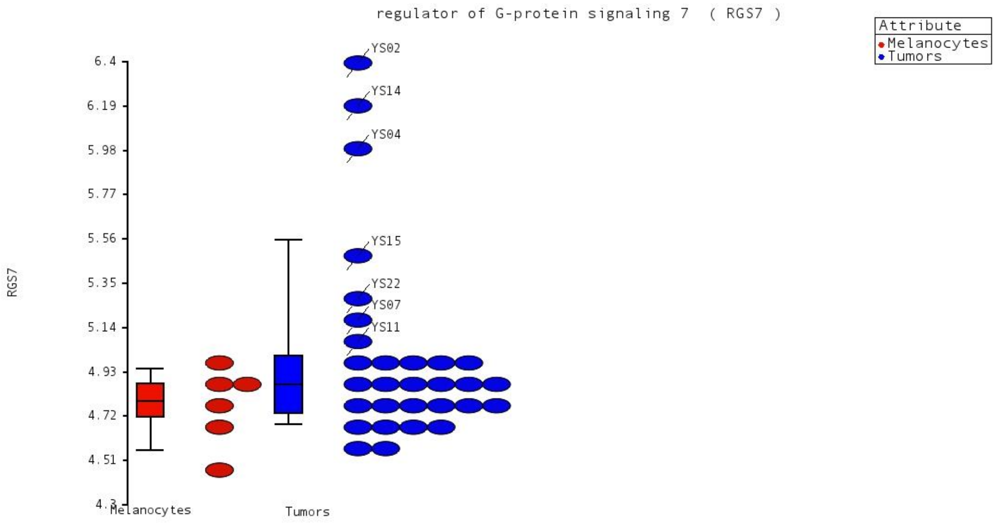

Supplementary Figure 3. RGS7 expression in melanocytes and melanoma samples. Relative log2 gene expression values Dot-plot distribution of the RGS7 gene in 30 melanoma tumors and 6 primary melanocytes. The expression value of the whole data set range between 3 and 15. The data was RMA (Robust Multichip Analysis) normalized.

R44

Human	MAQGNNYGQTSNGVADESPNMLVYRKMEDVIARMQDEKNGIPIRTVKSFLSKIPSVFSGSDIVQWLIKNLTIEDPVEALH
Mouse	MAQGNNYGQTSNGVADESPNMLVYRKMEDVIARMQDEKNGIPIRTVKSFLSKIPSVFSGSDIVQWLIKNLTIEDPVEALH
Cattle	MAQGNNYGQTSNGVADESPNMLVYRKMEDVIARMQDEKNGIPIRTVKSFLSKIPSVFSGSDIVQWLIKNLTIEDPVEALH
Rat	MA--TTAGPGTPGGRPRA-QILRLTKMERVVVSMQDPDQGVRIRSQRLLITVIPHAVTGSDIVEWLAQKFCVSEE-EALH
Zebrafish	MAKNNSLHPNSNGETDDSPNMLVYRKMEDVIARMQDEKNGIPIRTVKSFLSKIPSVFSGSDIVQWLLKNLCIEDQVEALH
Eagle	MAQGNNYGQSSNGVADESPNMLVYRKMEDVIARMQDEKNGIPIRTVKSFLSKIPSVFSGSDIVQWLTKNLSIEDPVEALH
Penguin	MAQGNNYGQSSNGVADESPNMLVYRKMEDVIARMQDEKNGIPIRTVKSFLSKIPSVFSGSDIVQWLTKNLSIEDPVEALH
Bird	MAQGNNYGQSSNGVADESPNMLVYRKMEDVIARMQDEKNGIPIRTVKSFLSKIPSVFSGSDIVQWLTKNLSIEDPVEALH
Monkey	MAQGNNYGQTSNGVADESPNMLVYRKMEDVIARMQDEKNGIPIRTVKSFLSKIPSVFSGSDIVQWLIKNLTIEDPVEALH
Rabbit	MAQGNNYGQSSNGVADESPNMLVYRKMEDVIARMQDEKNGIPIRTVKSFLSKIPSVFSGSDIVQWLIKNLTIEDPVEALH
Chimpanzee	MAQGNNYGQTSNGVADESPNMLVYRKMEDVIARMQDEKNGIPIRTVKSFLSKIPSVFSGSDIVQWLIKNLTIEDPVEALH
Gorilla	--------------------------MEDVIARMQDEKNGIPIRTVKSFLSKIPSVFSGSDIVQWLIKNLTIEDPVEALH
Killdear	MAQGNNYGQSSNGVADESPNMLVYRKMEDVIARMQDEKNGIPIRTVKSFLSKIPSVFSGSDIVQWLTKNLSIEDPVEALH

Supplementary Figure 4a. Comparison of conserved Arginine-44 of human RGS7 with its orthologs. The human RGS7 orthologs in species were compared using indicated NCBI accession numbers by COBALT algorithm (http://www.ncbi.nlm.nih.gov/tools/cobalt/cobalt.cgi). Conserved arginine at amino acid 44 in humans is shown and aligned with other species. The red color indicates highly conserved columns and blue indicates less conserved ones. The Conservation Setting can be used to select a threshold for determining which columns are colored in red.

```
                                                                           E383
Human        SKE--PSQQRVKRWGFGMDEALKDPVGREQFLKFLESEFSSENLRFWLAVEDLKKRPIKEVPSRVQEIWQEFLAPGAPSA
Mouse        SKE--PSQQRVKRWGFGMDEALKDPVGREQFLKFLESEFSSENLRFWLAVEDLKRRPIREVPSRVQEIWQEFLAPGAPSA
Cattle       SKE--PSQQRVKRWGFGMDEALKDPVGREQFLKFLESEFSSENLRFWLAVEDLKKRPIREVPSRVQEIWQEFLAPGAPSA
Rat          PMVavPTKLRVERWGFSFRELLDDPVGRAHFMDFLQKEFSVENLSFWEACEELRFGGQAQVPDLVDAVYQQFLAPSAACW
Zebrafish    SKE--PGQTRVRRWGFGIDEVLKDPVGREQFLKFLESEFSSENLRFWLAVQELKRRPIREVPTRVQEIWEEFLAAGAPSA
Eagle        SKE--PGQQRVKRWGFGMDEALKDPVGREQFLKFLESEFSSENLRFWLAVEDLKKRPIREVPARVQEIWQEFLAPGAPSA
Penguin      SKE--PGQQRVKRWGFGMDEALKDPVGREQFLKFLESEFSSENLRFWLAVEDLKKRPIREVPARVQEIWQEFLAPGAASA
Bird         SKE--PGQQRVKRWGFGMDEALKDPVGREQFLKFLESEFSSENLRFWLAVEDLKKRPIREVPARVQEIWQEFLAPGAPSA
Monkey       SKE--PSQQRVKRWGFGMDEALKDPVGREQFLKFLESEFSSENLRFWLAVEDLKKRPIKEVPSRVQEIWQEFLAPGAPSA
Rabbit       SKE--PSQQRVKRWGFGMDEALKDPVGREQFLKFLESEFSSENLRFWLAVEDLKKRPIREVPSRVQEIWQEFLAPGAPSA
Chimpanzee   SKE--PSQQRVKRWGFGMDEALKDPVGREQFLKFLESEFSSENLRFWLAVEDLKKRPIKEVPSRVQEIWQEFLAPGAPSA
Gorilla      SKE--PSQQRVKRWGFGMDEALKDPVGREQFLKFLESEFSSENLRFWLAVEDLKKRPIKEVPSRVQEIWQEFLAPGAPSA
Killdear     SKE--PGQQRVKRWGFGMDEALKDPVGREQFLKFLESEFSSENLRFWLAVEDLKKRPIREVPARVQEIWQEFLAPGAPSA
```

Supplementary Figure 4b. Comparison of Glutamic Acid-383 of human RGS7 with its orthologs. The human RGS7 orthologs in species were compared using indicated NCBI accession numbers by COBALT algorithm (http://www.ncbi.nlm.nih.gov/tools/cobalt/cobalt.cgi). Glutamic Acid at amino acid 383 in humans is shown and aligned with other species. The red color indicates highly conserved columns and blue indicates less conserved ones. The Conservation Setting can be used to select a threshold for determining which columns are colored in red.

R416

Human	INLDSKSYDKTTQNVKEPGRYTFEDAQEHIYKLMKS-------------------------------DSYPRFIRSSAYQ
Mouse	INLDSKSYDKTTQNVKEPGRYTFEDAQEHIYKLMKS-------------------------------DSYPRFIRSSAYQ
Cattle	INLDSKSYDKTTQNVKEPGRYTFEDAQEHIYKLMKS-------------------------------DSYPRFIRSSAYQ
Rat	VNIDSRTMERTLEGLPQSHCYVLDDAQLHIYMLMKKlgsaaslallepirvilsvlsiwflsrilqrDSYPRFLKSDMYK
Zebrafish	INVDSKSYDKTTQNVKDPGRYAFEDAQEHIYKLMKS-------------------------------DSYSRFIRSSAYQ
Eagle	INLDSKSYDKTTQNVKDPGRYTFEDAQEHIYKLMKS-------------------------------DSYPRFIRSSAYQ
Penguin	INLDSKSYDKTTQNVKDPGRYTFEDAQEHIYKLMKS-------------------------------DSYPRFIRSSAYQ
Bird	INLDSKSYDKTTQNVKEPGRYTFEDAQEHIYKLMKS-------------------------------DSYPRFIRSSAYQ
Monkey	INLDSKSYDKTTQNVKEPGRYTFEDAQEHIYKLMKS-------------------------------DSYPRFIRSSAYQ
Rabbit	INLDSKSYDKTTQNVKEPGRYTFEDAQEHIYKLMKS-------------------------------DSYPRFIRSSAYQ
Chimpanzee	INLDSKSYDKTTQNVKEPGRYTFEDAQEHIYKLMKS-------------------------------DSYPRFIRSSAYQ
Gorilla	INLDSKSYDKTTQNVKEPGRYTFEDAQEHIYKLMKS-------------------------------DSYPRFIRSSAYQ
Killdear	INLDSKSYDKTTQNVKDPGRYTFEDAQEHIYKLMKS-------------------------------DSYPRFIRSSAYQ

Supplementary Figure 4c. Comparison of conserved Arginine-416 of human RGS7 with its orthologs. The human RGS7 orthologs in species were compared using indicated NCBI accession numbers by COBALT algorithm (http://www.ncbi.nlm.nih.gov/tools/cobalt/cobalt.cgi). Conserved arginine at amino acid 416 in humans is shown and aligned with other species. The red color indicates highly conserved columns and blue indicates less conserved ones. The Conservation Setting can be used to select a threshold for determining which columns are colored in red.

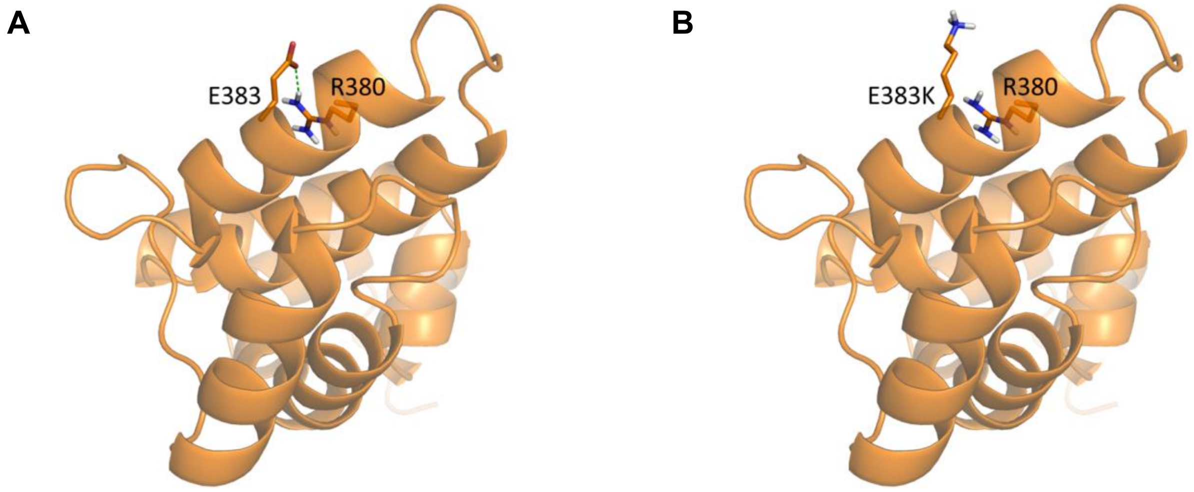

Supplementary Figure 5. The X-ray structure of the RGS domain (A) In the RGS7 RGS domain, residue E383 is positioned to form an H-bond with R380. This interaction is not likely in the E383K mutant (B).

A **B**

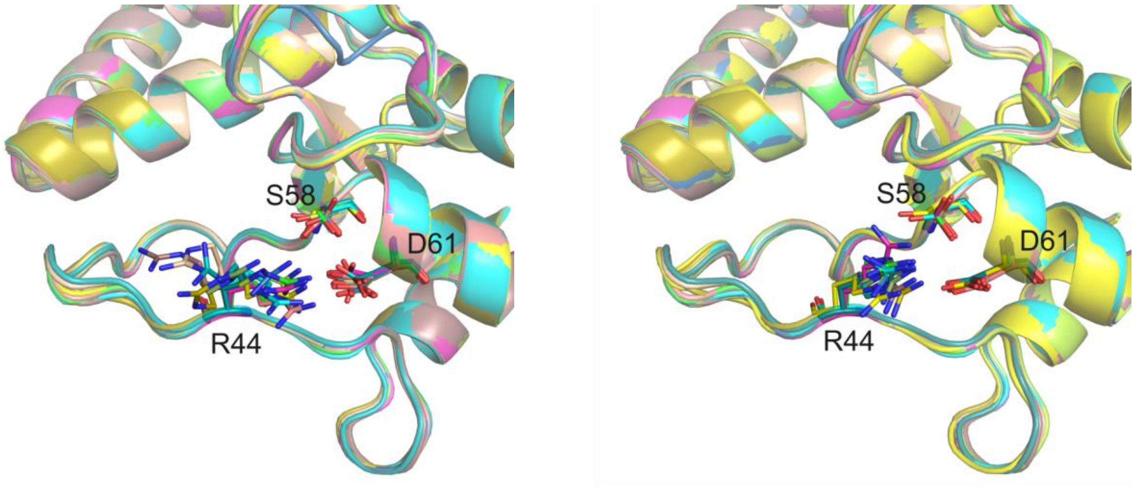

Supplementary Figure 6. Superimposition of the RGS7 3D structural models. Five models were generated by I-TASSER and Modeller, and one model with Prime and Phyre2, respectively. The picture shows the side chain orientation of R44 (A) before and (B) after side chain refinement.

```
                                                                                 D61
                                                                                  ↓
Human       MAQGNNYGQTSNGVADESPNMLVYRKMEDVIARMQDEKNGIPIRTVKSFLSKIPSVFSGSDIVQWLIKNLTIEDPVEALH
Mouse       MAQGNNYGQTSNGVADESPNMLVYRKMEDVIARMQDEKNGIPIRTVKSFLSKIPSVFSGSDIVQWLIKNLTIEDPVEALH
Cattle      MAQGNNYGQTSNGVADESPNMLVYRKMEDVIARMQDEKNGIPIRTVKSFLSKIPSVFSGSDIVQWLIKNLTIEDPVEALH
Rat         MA--TTAGPGTPGGRPRA-QILRLTKMERVVVSMQDPDQGVRIRSQRLLITVIPHAVTGSDIVEWLAQKFCVSEE-EALH
Zebrafish   MAKNNSLHPNSNGETDDSPNMLVYRKMEDVIARMQDEKNGIPIRTVKSFLSKIPSVFSGSDIVQWLLKNLCIEDQVEALH
Eagle       MAQGNNYGQSSNGVADESPNMLVYRKMEDVIARMQDEKNGIPIRTVKSFLSKIPSVFSGSDIVQWLTKNLSIEDPVEALH
Penguin     MAQGNNYGQSSNGVADESPNMLVYRKMEDVIARMQDEKNGIPIRTVKSFLSKIPSVFSGSDIVQWLTKNLSIEDPVEALH
Bird        MAQGNNYGQSSNGVADESPNMLVYRKMEDVIARMQDEKNGIPIRTVKSFLSKIPSVFSGSDIVQWLTKNLSIEDPVEALH
Monkey      MAQGNNYGQTSNGVADESPNMLVYRKMEDVIARMQDEKNGIPIRTVKSFLSKIPSVFSGSDIVQWLIKNLTIEDPVEALH
Rabbit      MAQGNNYGQSSNGVADESPNMLVYRKMEDVIARMQDEKNGIPIRTVKSFLSKIPSVFSGSDIVQWLIKNLTIEDPVEALH
Chimpanzee  MAQGNNYGQTSNGVADESPNMLVYRKMEDVIARMQDEKNGIPIRTVKSFLSKIPSVFSGSDIVQWLIKNLTIEDPVEALH
Gorilla     --------------------------MEDVIARMQDEKNGIPIRTVKSFLSKIPSVFSGSDIVQWLIKNLTIEDPVEALH
Killdear    MAQGNNYGQSSNGVADESPNMLVYRKMEDVIARMQDEKNGIPIRTVKSFLSKIPSVFSGSDIVQWLTKNLSIEDPVEALH
```

Supplementary Figure 7. Comparison of conserved Aspartic acid-61 of human RGS7 with its orthologs. The human RGS7 orthologs in species were compared using indicated NCBI accession numbers by COBALT algorithm (http://www.ncbi.nlm.nih.gov/tools/cobalt/cobalt.cgi). Conserved aspartic acid at amino acid 61 in humans is shown and aligned with other species. The red color indicates highly conserved columns and blue indicates less conserved ones. The Conservation Setting can be used to select a threshold for determining which columns are colored in red.

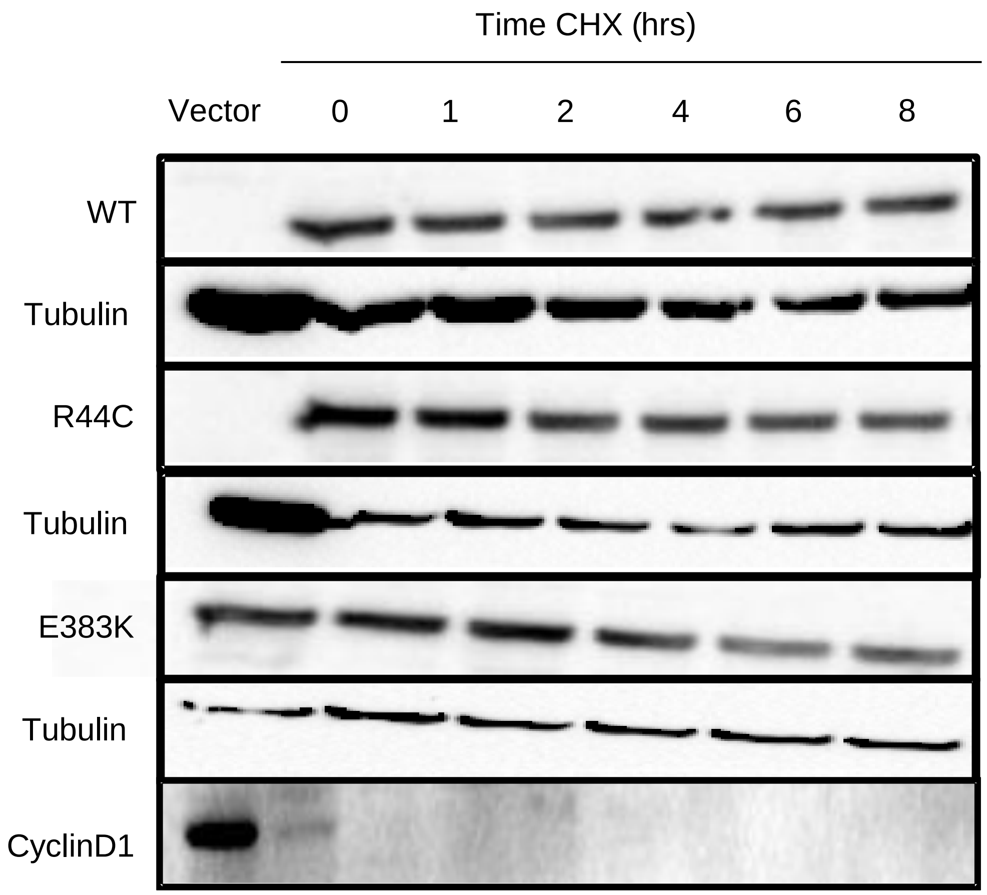

Supplementary Figure 8. Mutant RGS7 are less stable than wild-type RGS7. Cells expressing wild-type RGS7 or mutant RGS7 were treated with cycloheximide, collected at different time points and immunoblotted with anti-FLAG and anti-Tubulin. Cyclin D1 with a half-life of 20 minutes was used as a positive control. No vector is loaded in E383K. CHX, cycloheximide; WT, wild-type.

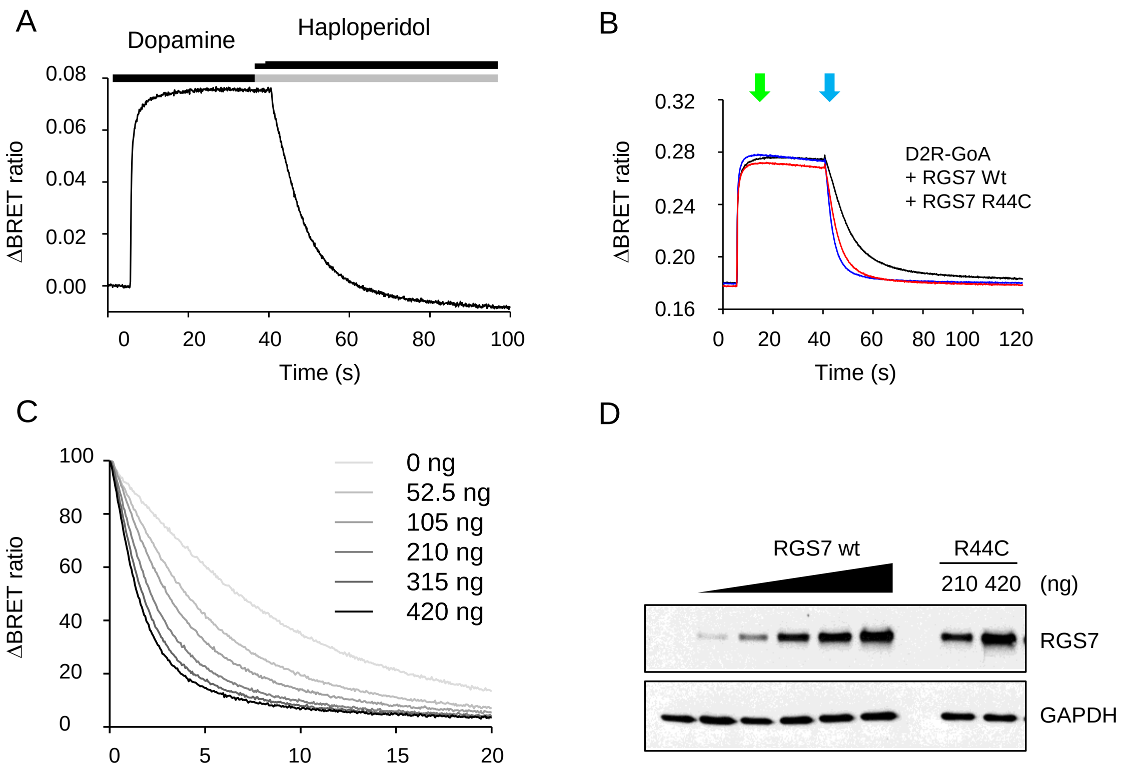

Supplementary Figure 9. Live-cell imaging platform for examining the activity of R44C mutant. (A) *In vitro* single BRET assay measuring the rate of GTP hydrolysis by the Gαo. Representative BRET response of cells reconstituted D2R-GoA signaling. Responses to sequential application of dopamine (100 μM) and haloperidol (100 μM) were recorded. Data are means of six wells. (B) HEK293T/17 cells were transiently transfected with dopamine D2 receptor (D2R), Gαo, and BRET sensors without RGS7 or with wild-type RGS7 or RGS7 R44C mutant. Responses to sequential application of dopamine (100 mM, *green arrow*) and haloperidol (100 mM, *cyan arrow*) (C) Time course of the normalized BRET responses recorded in a representative experiment. Individual data points show BRET values averaged from six replicates. (D) Western blot analysis of RGS7 expression levels of wild-type and mutant in transfected cells used in BRET assays.

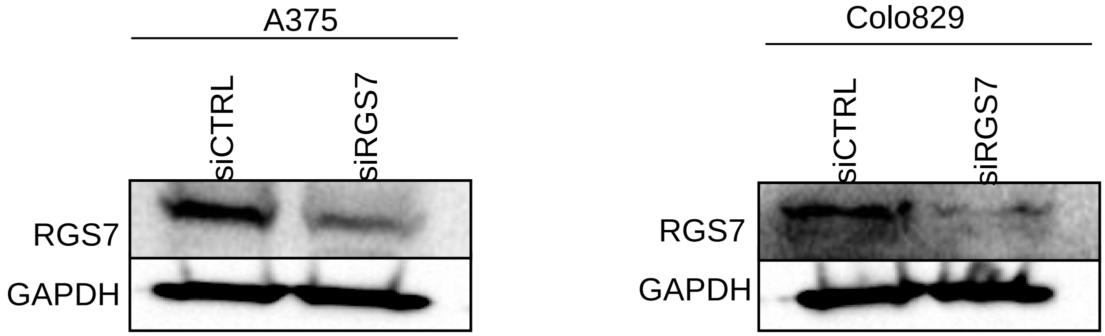

Supplementary Figure 10. Efficiency of siRNA depletion of endogenous *RGS7* in melanoma cells. A375 and colo829 cells were transfected with pool siRNA targeting *RGS7* or control for 72 hours. Detection of the RGS7 protein levels was done by western blotting. Lysates from the different clones were immunoblotted with anti-RGS7 7RC-1 antibody and were analyzed in parallel using anti-GAPDH.

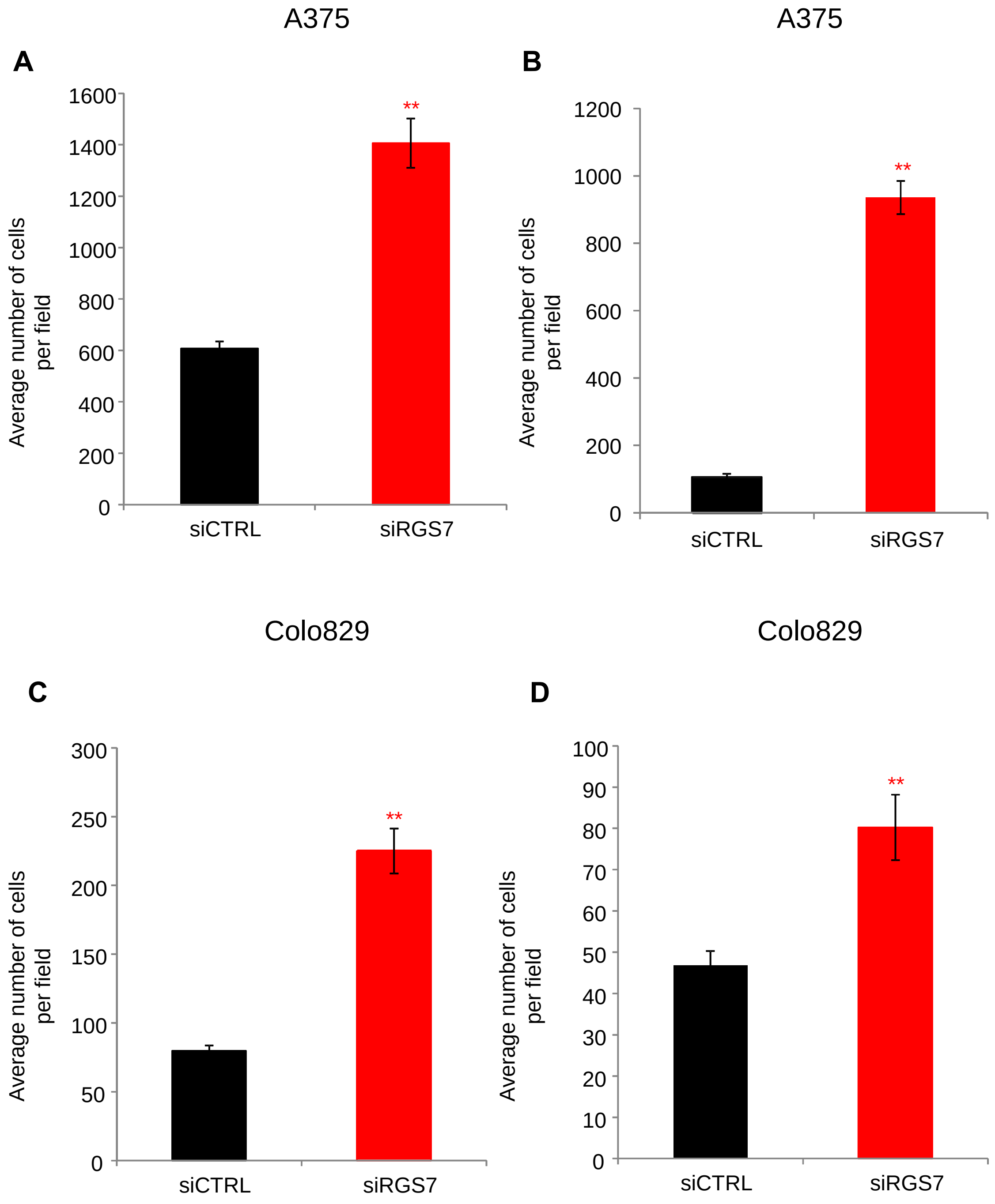

Supplementary Figure 11. RGS7 knockdown using pool siRNA increases migration and invasion of cells. A375 and colo829 cells were transiently transfected with pool siRNA that target *RGS7* or control for 72 hours. Clones expressing the indicated vectors were seeded in blind well chemotaxis chambers and assessed 16 h later for their ability to (A, C) migrate and (B, D) invade respectively. Stained filters were analyzed using a Nikon Eclipse TS100 microscope 4x lens and counted with ImageJ software. Quantification made from 2 independent experiments, each done in triplicates. ** $p<0.005$ for siRNA targeting *RGS7* vs vector (student's *t* tests); Error bars, S.D.

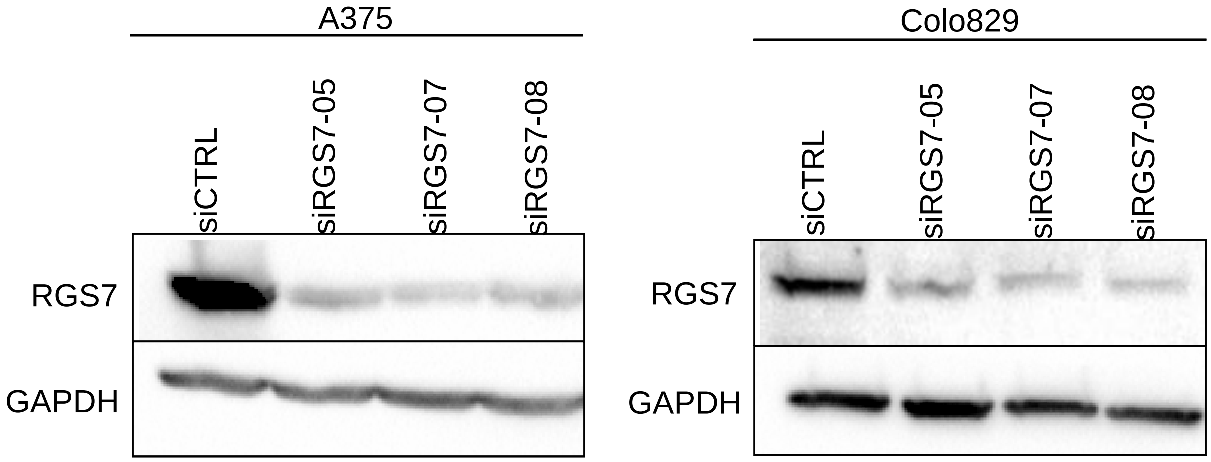

Supplementary Figure 12. Efficiency of individual siRNA depletion of endogenous *RGS7* in melanoma cells. A375 and colo829 cells were transfected with three individual siRNAs targeting *RGS7* or control for 72 hours. Detection of the RGS7 protein levels was done by western blotting. Lysates from the different clones were immunoblotted with anti-RGS7 7RC-1 antibody and were analyzed in parallel using anti-GAPDH.

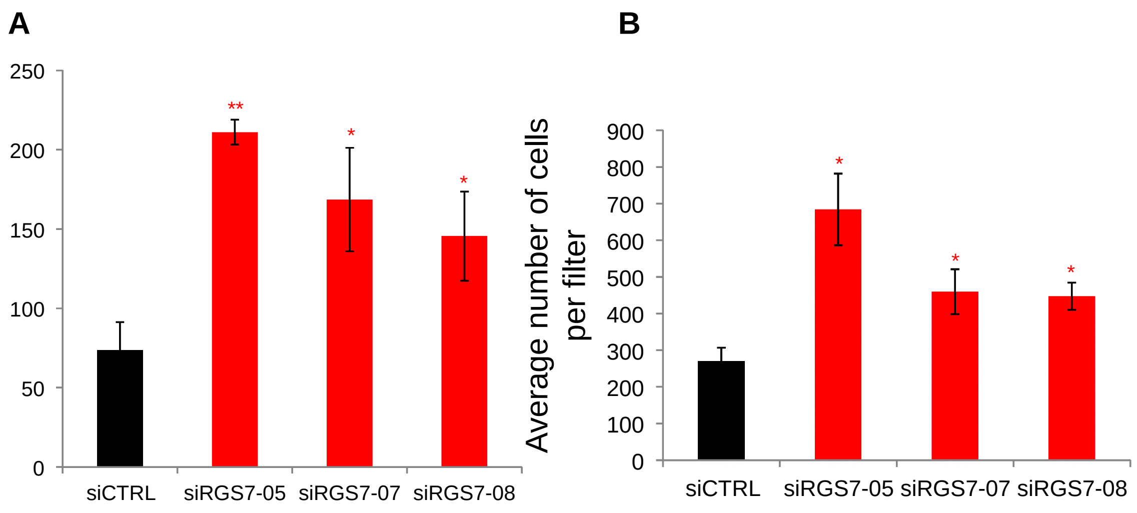

Supplementary Figure 13. RGS7 knockdown using single siRNAs increases migration and invasion of colo829. colo829 cells were transiently transfected with individual siRNAs that target *RGS7* or control for 72 hours. Clones expressing the indicated vectors were seeded in blind well chemotaxis chambers and assessed 16 h later for their ability to (A) migrate and (B) invade respectively. Stained filters were analyzed using a Nikon Eclipse TS100 microscope 4x lens and counted with ImageJ software. Quantification made from 2 independent experiments, each done in triplicates. . ** $p<0.005$, * $p<0.05$ for siRNA targeting *RGS7* vs control (student's *t* tests); Error bars, S.D**.**

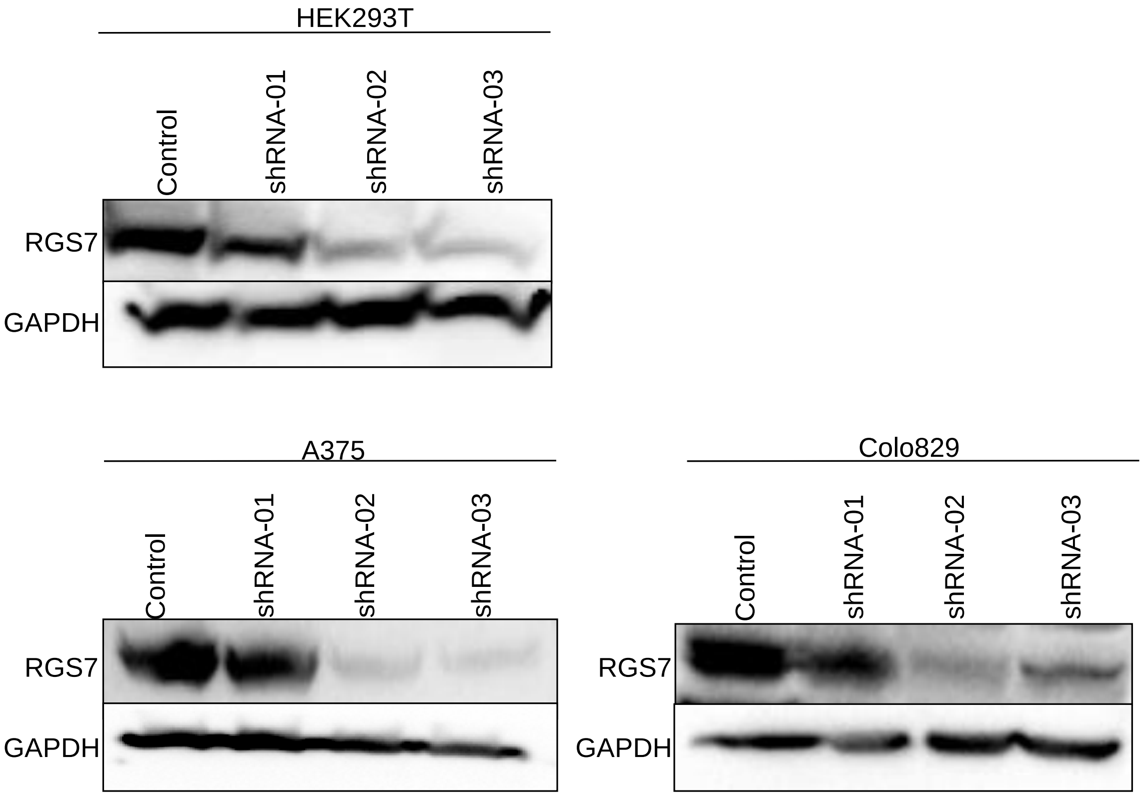

Supplementary Figure 14. Rescue of RGS7 expression using a non-targeting shRNA. cells were transiently transfected with wild-type RGS7 together with either vector control, 3'-UTR-targeting shRNA or two independent *RGS7*-specific shRNAs. Detection of the RGS7 protein levels was done by western blotting. Lysates from the different clones were immunoblotted with anti-FLAG antibody and were analyzed in parallel using anti-GAPDH

i

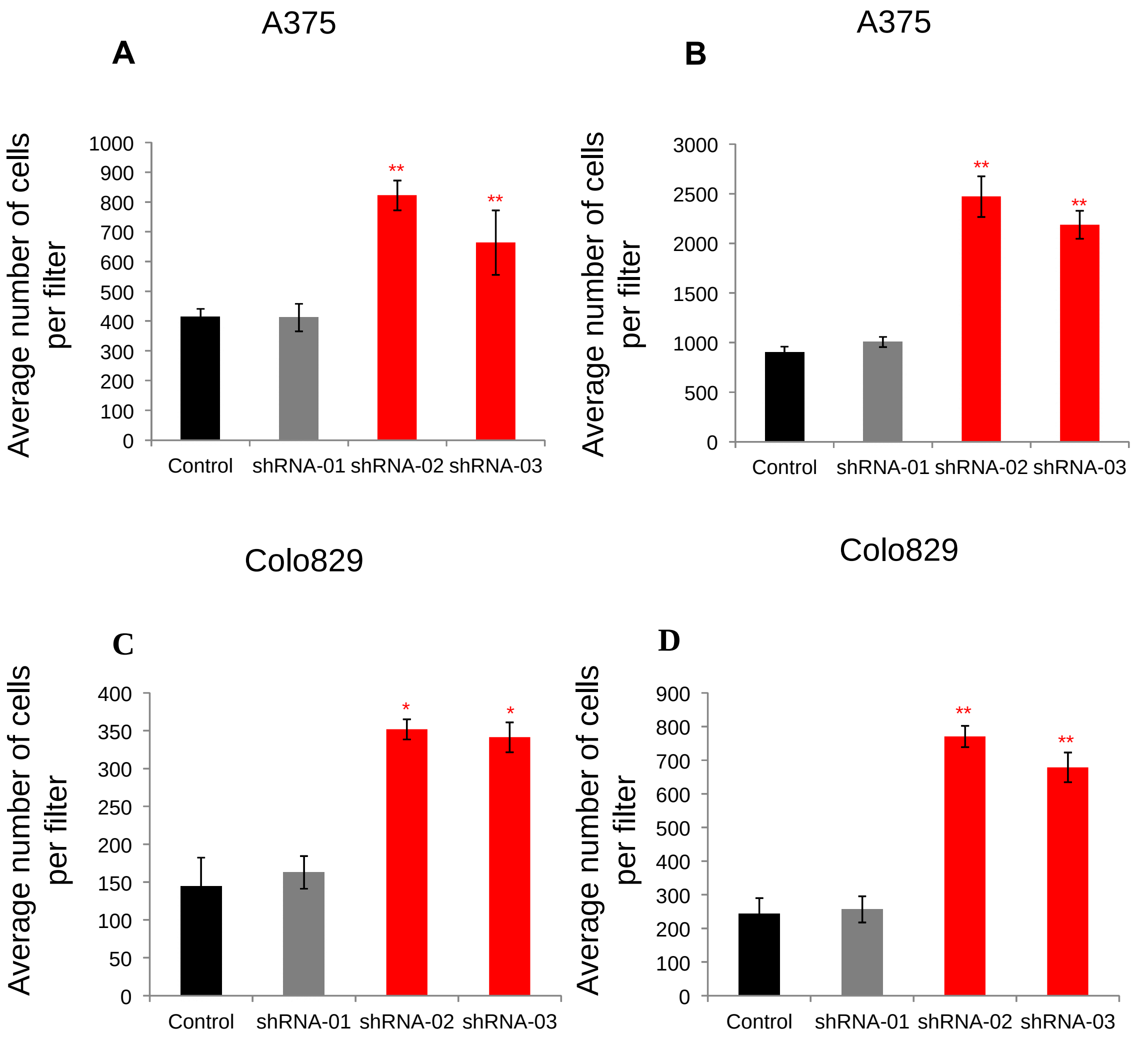

ii

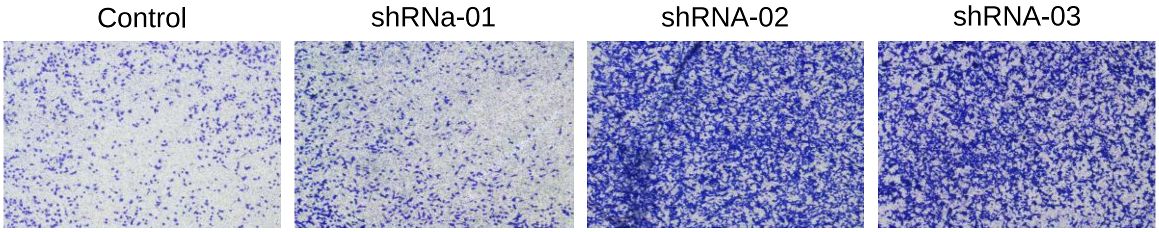

Supplementary Figure 15. Rescue of RGS7 knockdown migration and invasion phenotypes using a non-targeting RGS7 shRNA. Cells expressing wild-type RGS7 were stably infected with vector control, 3' UTR-targeting shRNA and two independent *RGS7*-specific shRNAs. Clones expressing the indicated vectors were seeded in blind well chemotaxis chambers and assessed 16 h later for their ability to (A, C) migrate and (B, D) invade respectively. Stained filters were analyzed using a Nikon Eclipse TS100 microscope 4x lens and counted with ImageJ software. Quantification made from 2 independent experiments, each done in triplicates. ** $p<0.005$, *$p<0.05$ for vector vs shRNAs targeting RGS7

(student's *t* tests); Error bars, S.D. Representative images of invaded A375 cells are shown in ii.

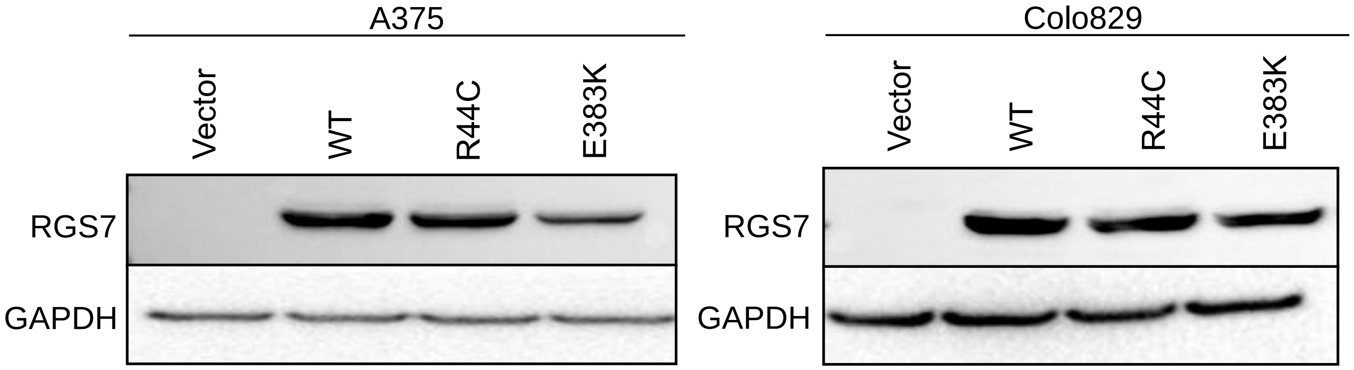

Supplementary Figure 16. Expression of wild-type and mutant RGS7 in melanoma cells. Detection of the RGS7 protein levels in A375 and colo829 melanoma cells stably infected with wild-type, R44C, E383K or vector alone. Lysates from the different clones were immunoblotted with FLAG antibody and were analyzed in parallel using anti-GAPDH for normalization. WT, wild-type.

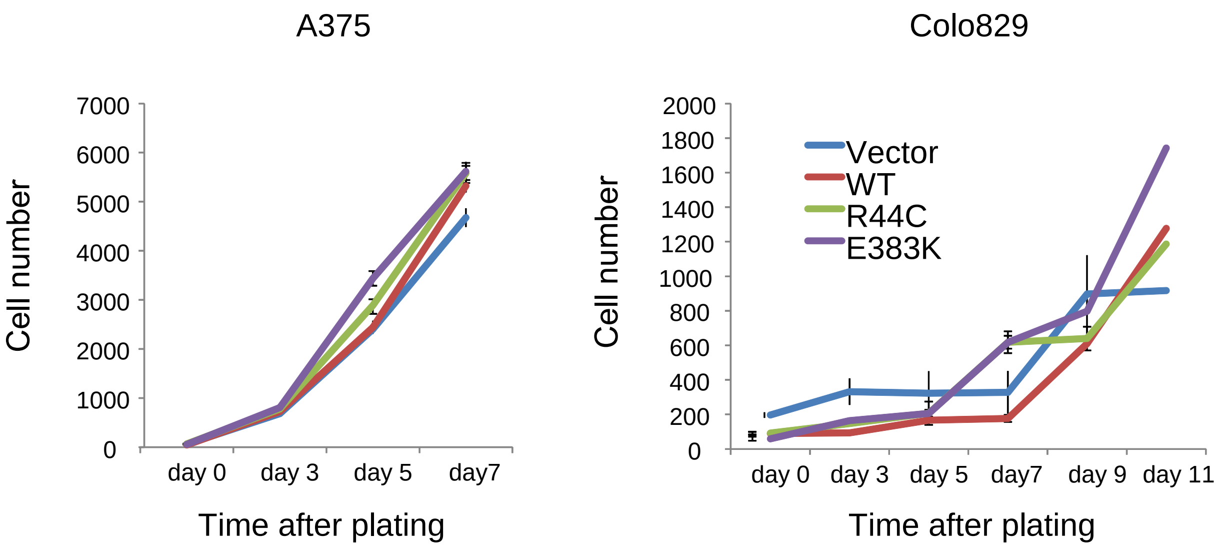

Supplementary Figure 17. Effects of wild-type and mutant RGS7 on cell growth. No difference is found between wild-type and mutant on proliferation. A375 and colo829 pooled clones expressing wild-type, R44C, E383K or vector alone were seeded in full serum in 96-well plates and incubated over a 7 day period for A375 and 11 day period for colo829. Plates were harvested and average cell number at each time point was measured by assessing DNA content using SYBR Green I. Error bars, standard deviation (S.D); WT, wild-type.

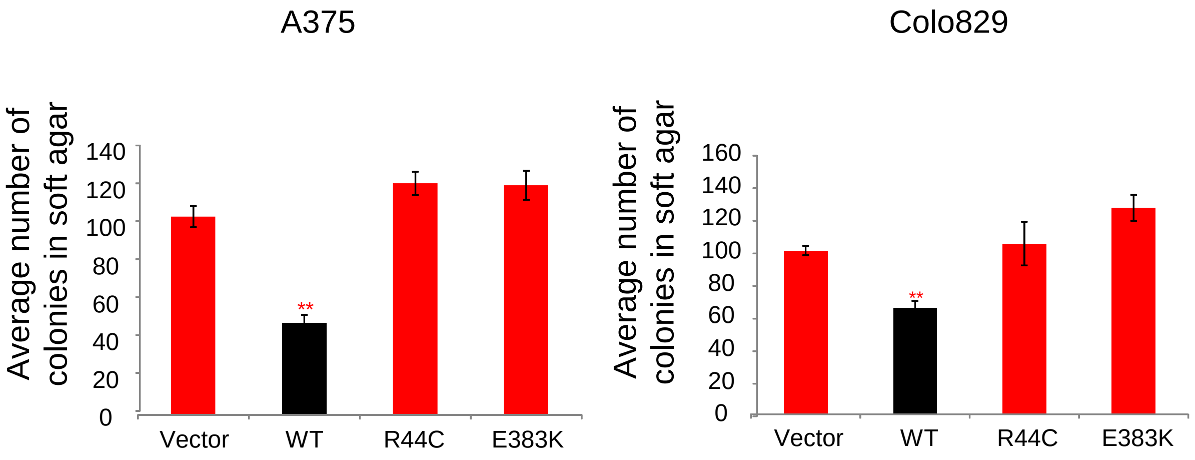

Supplementary Figure 18. Effects of wild-type and mutant RGS on anchorage-independent growth. A375 and colo829 pooled clones expressing mutant RGS7 R44C and E383K fail to decrease cell anchorage- independent growth compared to cells expressing wild-type RGS7. Anchorage-independent growth of A375 and colo829 pooled RGS7 clones expressing wild-type, R44C, E383K or vector alone were assessed by measuring colony formation in soft agar in medium with 10% serum in two independent experiments with four replicates each 14 days after plating. ** $p<0.05$ for wild-type RGS7 versus vector (student's *t* tests). Error bars, standard deviation (S.D); WT, wild-type.

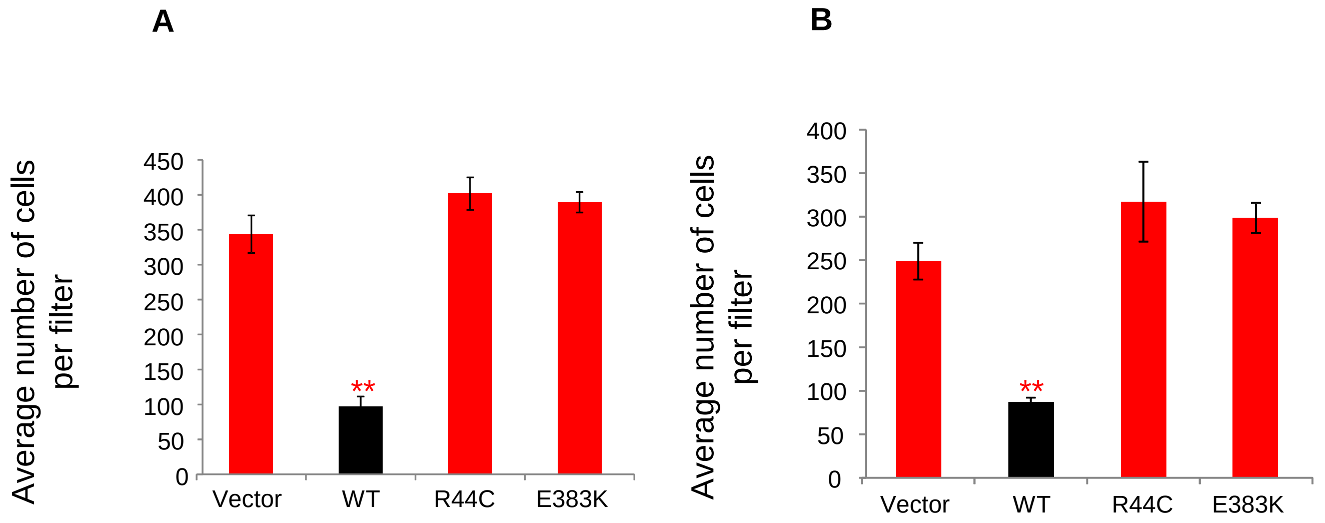

Supplementary Figure 19. Effects of wild-type and mutant RGS7 on cell migration and invasion in colo829. (A) The migration ability of colo829 expressing wild-type and mutant RGS7 were assessed by seeding colo829 clones expressing the indicated vectors in blind well chemotaxis chambers and assessed for their ability to migrate 16 h later. (B) The invasion ability of colo829 expressing wild-type and mutant RGS7 were assessed by seeding colo829 clones expressing the indicated vectors in blind well chemotaxis chambers with matrigel and assessed for their ability to invade 16 h later. Filters were stained and images were taken using a Nikon Eclipse TS100 microscope 4x lens and counted with ImageJ software. Quantification made from 2 independent experiments, each done in triplicates. ** $p<0.005$ for wild-type RGS7 versus vector (student's *t* tests). Error bars, standard deviation (S.D); WT, wild-type.

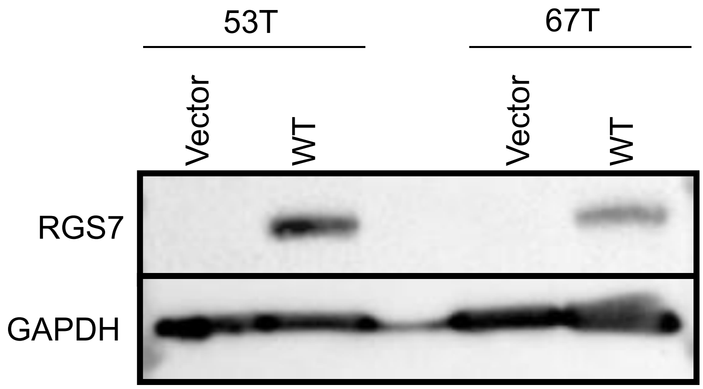

Supplementary Figure 20. Expression of wild-type RGS7 in melanoma cells. Detection of the RGS7 protein levels in 53T and 67T melanoma cells stably infected with wild-type or vector alone. Lysates from the different clones were immunoblotted with anti-FLAG antibody. We analyzed the lysates in parallel using anti-GAPDH for normalization; WT, wild-type.

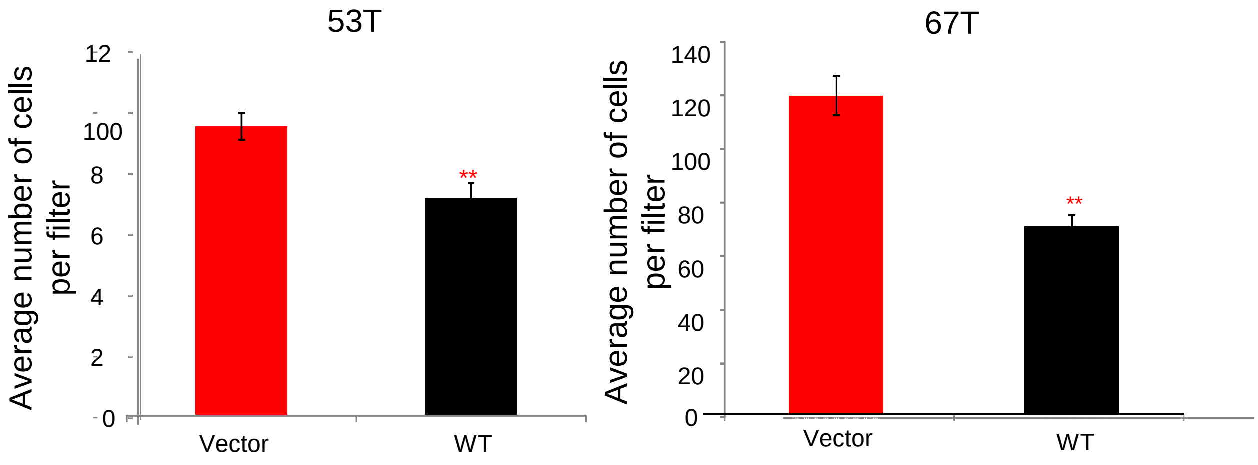

Supplementary Figure 21. Effects of wild-type RGS7 on cell migration in melanoma cell types. The migration ability of 53T and 67T expressing wild-type were assessed by seeding 53T and 67T clones expressing the indicated vectors in blind well chemotaxis chambers and assessed for their ability to migrate 20 h later. Filters were stained and images were taken using a Nikon Exlipse TS100 microscope 4x lens and counted with ImageJ software. Quantification made from 2 independent experiments, each done in triplicates. ** $p<0.005$ for wild-type RGS7 versus vector (student's *t* tests); Error bars, standard deviation (S.D); WT, wild-type.

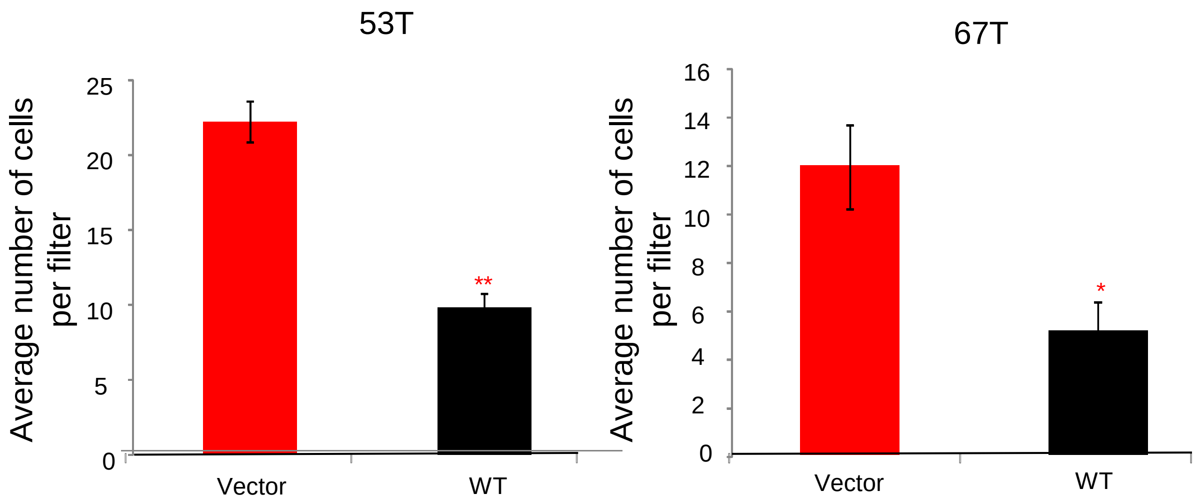

Supplementary Figure 22. Effects of wild-type RGS7 on cell invasion in melanoma cell types. The invasion ability of 53T and 67T expressing wild-type RGS7 were assessed by seeding 53T and 67T clones expressing the indicated vectors in blind well chemotaxis chambers with matrigel and assessed for their ability to invade 20 h later. Filters were stained and images were taken using a Nikon Exlipse TS100 microscope 4x lens and counted with ImageJ software. Quantification made from 2 independent experiments, each done in triplicates. ** $p<0.05$, * $p<0.5$ for wild-type RGS7 versus vector (student's *t* tests); Error bars, standard deviation (S.D); WT, wild-type.

A

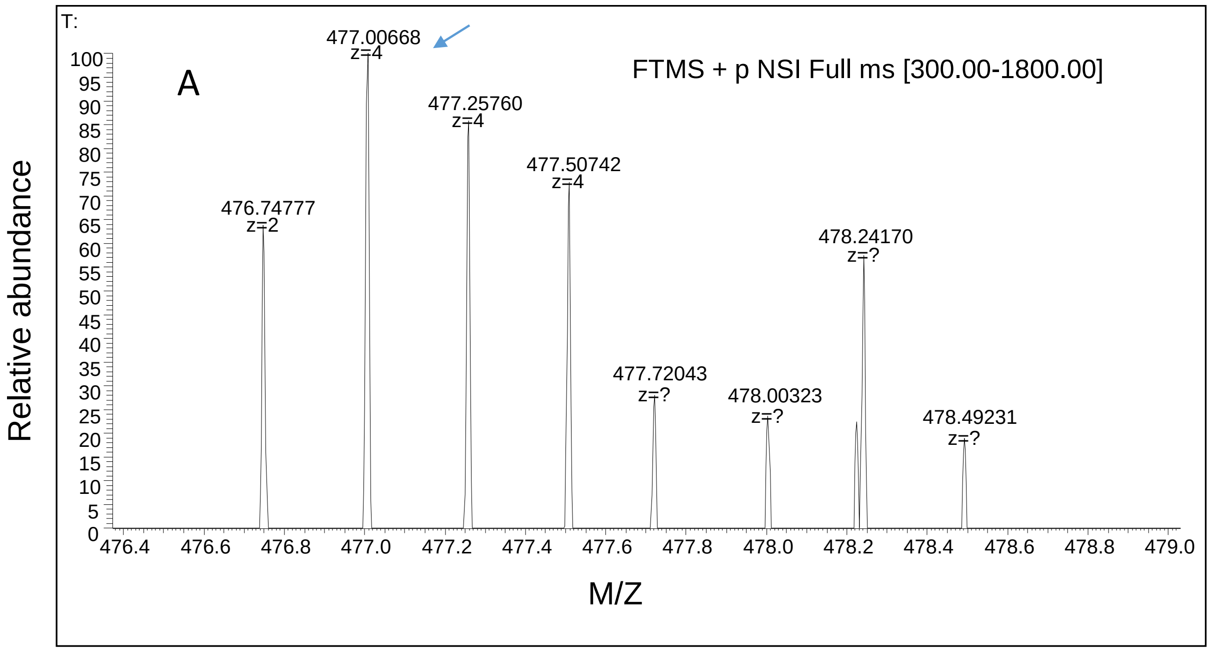
T:
A
FTMS + p NSI Full ms [300.00-1800.00]
476.74777
z=2
477.00668
z=4
477.25760
z=4
477.50742
z=4
477.72043
z=?
478.00323
z=?
478.24170
z=?
478.49231
z=?
Relative abundance
M/Z

B

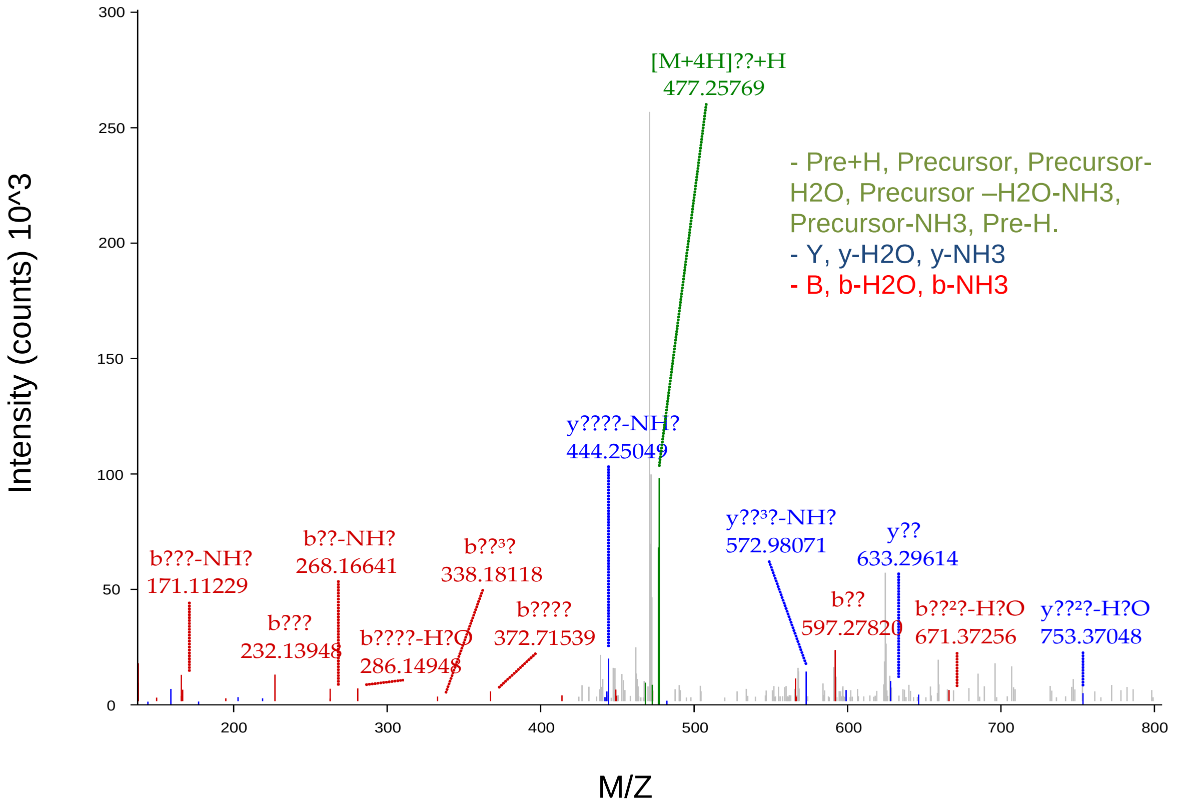
[M+4H]??+H
477.25769
- Pre+H, Precursor, Precursor-H2O, Precursor –H2O-NH3, Precursor-NH3, Pre-H.
- Y, y-H2O, y-NH3
- B, b-H2O, b-NH3
y????-NH?
444.25049
b???-NH?
171.11229
b??-NH?
268.16641
b???
232.13948
b????-H?O
286.14948
b??³?
338.18118
b????
372.71539
y??³?-NH?
572.98071
y??
633.29614
b??
597.27820
b??²?-H?O
671.37256
y??²?-H?O
753.37048
Intensity (counts) 10^3
M/Z

Supplementary Figure 23. Evaluation of S-S bond formation in RGS7 R44C/ V56C using Mass spectroscopy analysis. (A) MS spectrum of the peptide NGIPICTVKSFLSKIPSC with S-S bond (C6-Dehydro (-1.007 Da), C18-Dehydro (-1.007 Da)). (B) HCD spectrum of the peptide.

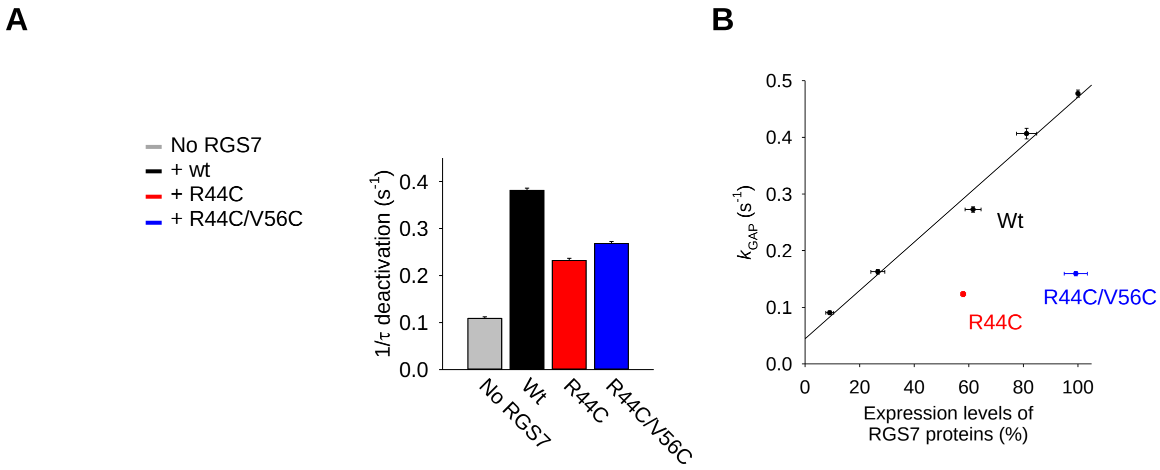

Supplementary Figure 24. The effect of the rescue mutant (R44C/V56C) on its catalytic activity. (a) Time course of normalized BRET responses recorded in a representative experiment. *Left.* The deactivation phase after antagonist application is shown. Wild-type RGS7 or mutant were transfected at equal amount of cDNA (210 ng) together with dopamine D2 receptor, Gαo, and BRET sensor pair. *Right*. Quantification of the exponential decay kinetics of the response. BRET values were averaged from four or six replicates. *, $P < 0.0001$. (b) Correlation analysis between expression levels of RGS7 and activity.

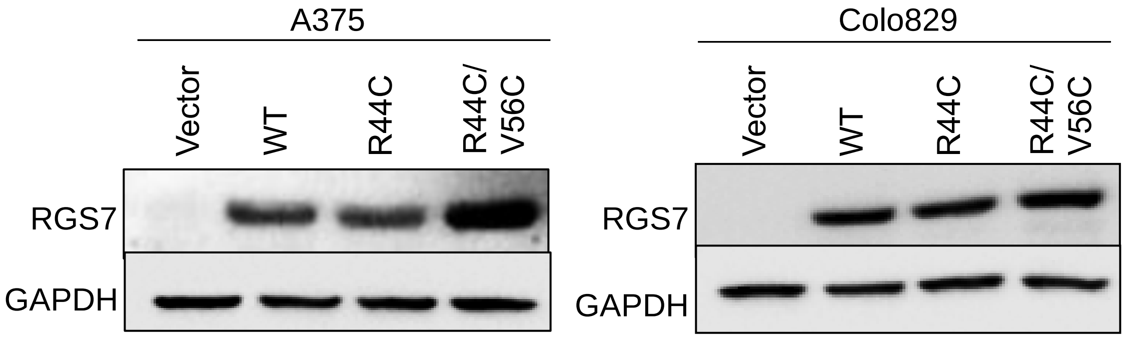

Supplementary Figure 25. Expression of wild-type and mutant RGS7 in melanoma cells. Detection of the RGS7 protein levels in A375, Colo829 melanoma cells transiently transfected with an empty vector, wild-type and the mutant RGS7. Lysates from the different clones were immunoblotted with anti-FLAG antibody and were analyzed in parallel using anti-GAPDH for normalization. WT, wild-type.

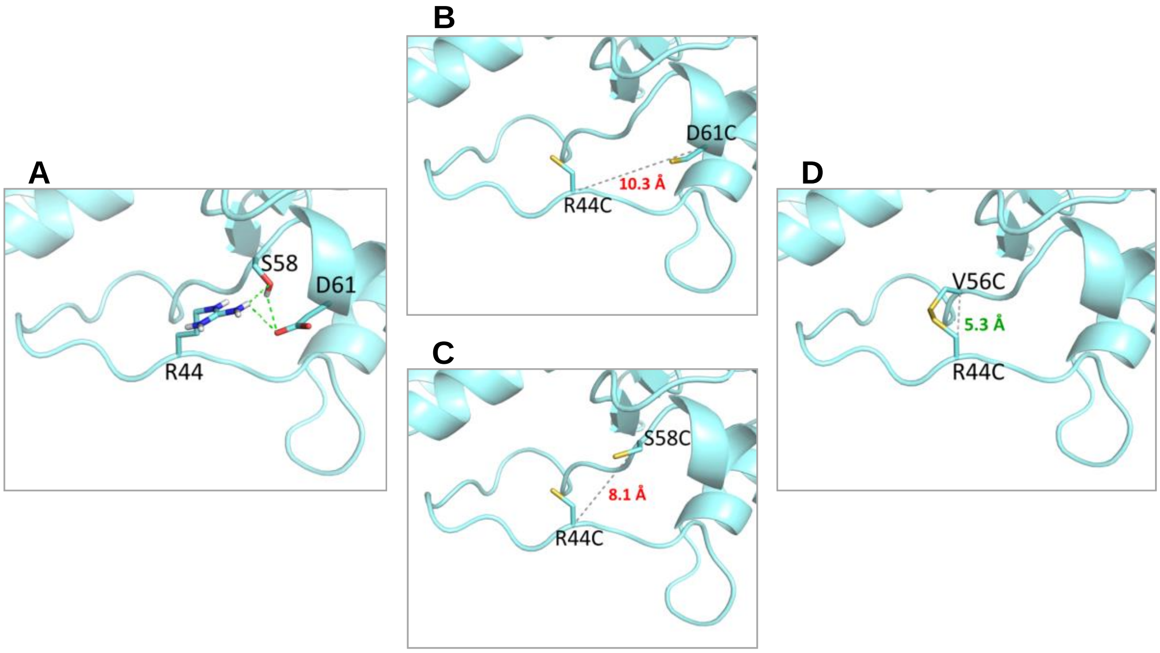

Supplementary Figure 26. Design of disulfide bridge in the R44C mutant RGS7. (A) Wild-type *RGS7*. (B) A D61C point mutation relative to the R44C mutation. (C) A S58C point mutation relative to the R44C mutation. (D) A V56C point mutation relative to the R44C mutation. Green dashed lines show H-bond interactions and gray dashed lines represent Cα-Cα distances. Cα-Cα distances in red are too large while green ones are suitable for disulfide bond formation in the crystallographic conformation of the loop.

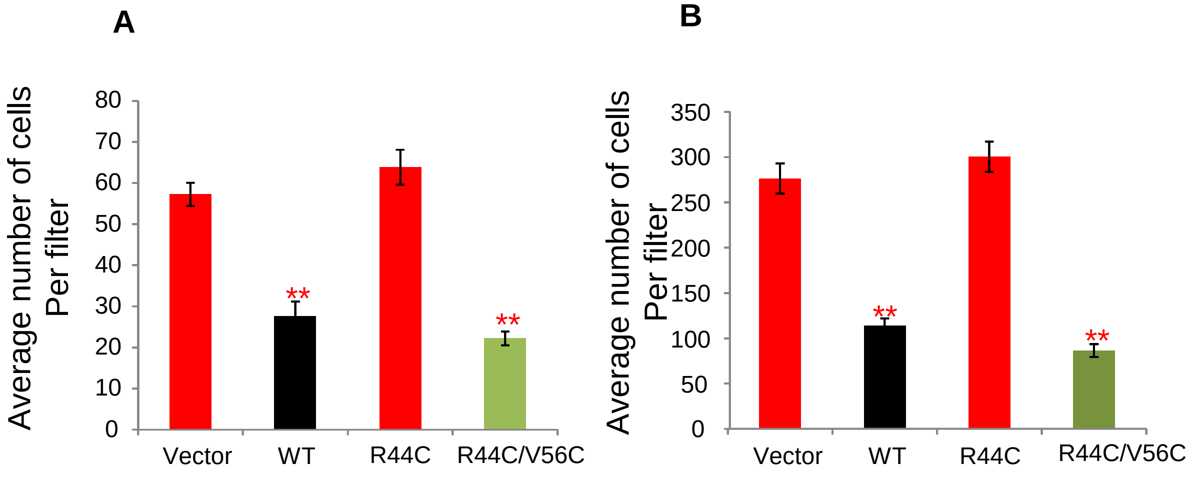

Supplementary Figure 27. Effects of wild-type and mutant RGS7 on cell migration and invasion in colo829. (A) The migration ability of colo829 expressing wild-type, mutant R44C and R44C/V56C were assessed by seeding colo829 clones expressing the indicated vectors in blind well chemotaxis chambers and assessed for their ability to migrate 16 h later. (B) The invasion ability of colo829 expressing wild-type, mutant R44C and R44C/V56C were assessed by seeding colo829 clones expressing the indicated vectors in blind well chemotaxis chambers with matrigel and assessed for their ability to invade 16 h later. Filters were stained and images were taken using a Nikon Eclipse TS100 microscope 4x lens and counted with ImageJ software. Quantification made from 2 independent experiments, each done in triplicates. ** $p<0.005$ for wild-type RGS7 versus vector and for R44C/V56C versus vector (student's *t* tests). Error bars, standard deviation (S.D); WT, wild-type.

Supplementary Tables

Supplementary Table 1. Somatic mutations identified in 501 melanoma whole exomes and whole genomes. Attached individually in Excel format.

Supplementary Table 2. Mutations recurring at 4+ melanoma samples. Attached individually in Excel format.

Supplementary Table 3. SIFT scores for non-synonymous mutations occurring in *RGS7.* Attached individually in Excel format.

Protein_Change	Genome_Change	Sift Score	Mutation type based on sift score
p.A150V	g.chr1:241033356G>A	0.052	Tolerated
p.D103N	g.chr1:241099926C>T	0.577	Tolerated
p.D104N	g.chr1:241099923C>T	0	Damaging
p.D127N	g.chr1:241094023C>T	0.001	Damaging
p.D182N	g.chr1:241031952C>T	0.018	Damaging
p.D236N	g.chr1:240979694C>T	0.199	Tolerated
p.D36N	g.chr1:241262035C>T	0.039	Damaging
p.E124K	g.chr1:241094032C>T	0.005	Damaging
p.E255K	g.chr1:240979637C>T	0.947	Tolerated
p.E288K	g.chr1:240977012C>T	0.012	Damaging
p.E292K	g.chr1:240977000C>T	0.011	Damaging
p.E315K	g.chr1:240976931C>T	0.111	Tolerated
p.E358K	g.chr1:240975228C>T	0	Damaging
p.E37K	g.chr1:241262032C>T	0.041	Damaging
p.E383K	g.chr1:240969562C>T	0.092	Tolerated
p.G455E	g.chr1:240964804C>T	1	Tolerated
p.H97Y	g.chr1:241099944G>A	0.002	Damaging
p.I184M	g.chr1:241031944A>C	0.217	Tolerated
p.I31L	g.chr1:241262050T>G	0.053	Tolerated
p.K452N	g.chr1:240966207T>A	0.002	Damaging
p.L189F	g.chr1:241031931G>A	0.007	Damaging
p.M334I	g.chr1:240975298C>G	0.573	Tolerated
p.M430I	g.chr1:240966273C>T	0.003	Damaging
p.P42S	g.chr1:241262017G>A	0.055	Tolerated
p.P436L	g.chr1:240966256G>A	0.005	Damaging
p.P93S	g.chr1:241099956G>A	0.018	Damaging
p.Q111*	g.chr1:241099902G>A	NA	Damaging
p.Q382*	g.chr1:240969565G>A	NA	Damaging
p.Q423*	g.chr1:240969442G>A	NA	Damaging
p.R194K	g.chr1:241031915C>T	0.003	Damaging
p.R273W	g.chr1:240978044G>A	0.022	Damaging
p.R361K	g.chr1:240975218C>T	0.344	Tolerated
p.R416Q	g.chr1:240969462C>T	0.02	Damaging
p.R44C	g.chr1:241262011G>A	0	Damaging
p.R460C	g.chr1:240964790G>A	0.002	Damaging
p.S18L	g.chr1:241519024G>A	0.036	Damaging
p.S474F	g.chr1:240939506G>A	0.177	Tolerated
p.S58F	g.chr1:241261968G>A	0.001	Damaging
p.T112_splice	g.chr1:241094069C>T	0	Damaging
p.V327I	g.chr1:240975321C>T	0.068	Tolerated

Abbreviations: NA, unaviable

Supplementary Table 4. Z-score RNA seq expression in 479 TCGA melanoma samples in Excel format.

Supplementary Table 5. Mutation data for the main melanoma drivers of melanoma patients on which RGS7 immunohistochemistry (IHC) was performed in Excel format.

Supplementary Table 6. Cell line genotypes used in the study

Cell line/Gene	NRAS	BRAF	RGS7
A375	WT	p.V600E	WT
Colo829	WT	p.V600E	WT

Supplementary Table 7. Primers used for PCR amplification and sequencing of RGS7

Primer Coverage	Forward	Reverse	Sequencing primer
1	TGTAAAACGACGGCCAGTGAGGCATTGAGACGGAAGAG	TTTCAGCCATTGAACAAGAATG	TGTAAAACGACGGCCAGT
2	TGTAAAACGACGGCCAGTTGTTGTAATATAAACTTCTTTCCAAACT	TGGAATTAATGATGTTGTCAATAGG	TGTAAAACGACGGCCAGT
3	TGTAAAACGACGGCCAGTTGCAAAGCATGAACATCTG	GAGTCAACATTACCCACTTCAGG	TGTAAAACGACGGCCAGT
4	TGTAAAACGACGGCCAGTTGAGGTGGGGTCATCATTAGA	TCCTTTGAACACAGTCAGTACCA	TGTAAAACGACGGCCAGT
5	TGTAAAACGACGGCCAGTATACGTTGGACAGCCTTTGC	TGGCACTGGGGTCAGAAT	TGTAAAACGACGGCCAGT
6	TGTAAAACGACGGCCAGTTGACTAATTGCACCTCTAATTTTGG	TGCTGTGGGTGGACTGATTA	TGTAAAACGACGGCCAGT
7	TGTAAAACGACGGCCAGTCATGTCTTCCAGCAGGAGTG	ACTGCACCCAGCAAAATCTC	TGTAAAACGACGGCCAGT
8	TGTAAAACGACGGCCAGTTGGAGTCACTTACAAATCAGCA	TGCTCTCAAATTTTATCACATGC	TGTAAAACGACGGCCAGT
9	TGTAAAACGACGGCCAGTCTTCTCTCATCAGTATCAGGTTTTCT	ACGTATTGACACCACTTTCACA	TGTAAAACGACGGCCAGT
10	TGTAAAACGACGGCCAGTAGCAAGAGAGTAAGCAAGCGATA	GCCAATATGAAGATGAGCTGAA	TGTAAAACGACGGCCAGT
11	TGTAAAACGACGGCCAGTAGAAAAGGCTGTGGGCCTTA	AAGATGCTAAAAACTGTGATGACC	TGTAAAACGACGGCCAGT
12	TGTAAAACGACGGCCAGTATAGTTGCAGAGGGCACCAT	GAGCTACCAAAAGCAGTGTTGA	TGTAAAACGACGGCCAGT
13	TGTAAAACGACGGCCAGTTCATCACACAATTTAGCTTTCACA	GCATGTTTGGGCATGTCAG	TGTAAAACGACGGCCAGT
14	TGTAAAACGACGGCCAGTTGGCAGAATTAAGGATGTGAAA	GGTTTGACTTGAGAGGGGATA	TGTAAAACGACGGCCAGT
15	TGTAAAACGACGGCCAGTCAAACTAACCTAGAGCAGGGTCA	TCTACTTGCTTTTGCACTGATTTC	TGTAAAACGACGGCCAGT
16	TGTAAAACGACGGCCAGTCTTCAGTGTGTTTCCCCACAT	TCACCAAGTTCAGATGCAAGT	TGTAAAACGACGGCCAGT

Supplementary Table 8. SiRNAs used in the study

J-015720-05	GAAGGAACCUGGACGAUAC
J-015720-06	UUAGAUAGACAUCGGUUAA
J-015720-07	GACUGGAGCUCGCAGACUA
J-015720-08	ACACAGAUUAUGCCGUUUA

Supplementary Table 9. ShRNAs used in the study

shRNA-01	TRCN0000014323	ATTGAGCTACAAAGTGTGTGC
shRNA-02	TRCN0000014324	TAAACCATAGACAGACTTCCG
shRNA-03	TRCN0000014325	TTCAAATGTGTATCGTCCAGG
